# aVeRsive tension: A new virtual reality paradigm to assess emotional arousal in adolescent and young adult patients with symptoms of borderline personality disorder

**DOI:** 10.1016/j.ijchp.2025.100583

**Published:** 2025-05-05

**Authors:** Sabrina Mittermeier, Klara Gregorova, Christopher Goettfert, Christian Merz, Martin Weiß, Jana Krauss, Sarah Franke, Andrea Reiter, Carolin Wienrich, Arne Buerger

**Affiliations:** a Department of Child and Adolescent Psychiatry, Psychosomatics and Psychotherapy, Centre of Mental Health, University Hospital of Wuerzburg, Wuerzburg, Germany; b German Centre of Prevention Research in Mental Health, University of Wuerzburg, Wuerzburg, Germany; c Institute for Human-Computer-Media, Psychology of Intelligent Interactive Systems, University of Wuerzburg, Wuerzburg, Germany; d Department of Psychology I – Clinical Psychology and Psychotherapy, University of Wuerzburg, Wuerzburg, Germany; e Department of Psychology I – Psychotherapy and Intervention Psychology, University of Wuerzburg, Wuerzburg, Germany

**Keywords:** Borderline personality disorder, Virtual reality, Emotional arousal, Adolescents, Social stress, Trier social stress test, Cyberball

## Abstract

**Background:**

High emotional arousal (EA) is a core feature of borderline personality disorder (BPD). While virtual reality (VR) has shown promise in treating emotion-based disorders, research on VR applications for BPD remains limited, especially in adolescence. This study aimed to validate a novel VR-based *aVeRsive tension* paradigm for assessing EA in adolescents and young adults with BPD symptoms.

**Methods:**

In a multimodal study, we investigated the validity of aVeRsive tension: We surveyed 62 patients with BPD symptoms and 62 healthy controls (HC) aged 13–25 years who completed two VR sessions (stress/control condition). Each session included an adapted Trier Social Stress Test (TSST) followed by a cyberball paradigm. Subjective EA ratings and physiological measurements were taken during the sessions.

**Results:**

The BPD group showed significantly higher EA levels compared to HC across both conditions. While both groups exhibited peak EA after TSST, HC demonstrated EA reduction during Cyberball in both conditions. The BPD group maintained elevated EA levels in the stress condition. Physiological data partially supported these findings, with the BPD group showing higher heart rates, particularly during Cyberball in the stress condition.

**Discussion:**

The *aVeRsive tension* paradigm successfully discriminated between BPD and HC groups, capturing both subjective and physiological responses. The sustained EA in the BPD group during stress conditions aligns with characteristic emotion dysregulation patterns. While task-specific effects were observed, with TSST eliciting stronger responses than Cyberball, the paradigm effectively simulated real-life stressors in a controlled VR environment.

**Conclusion:**

This study validates the *aVeRsive tension* protocol as a promising tool for assessing EA in adolescents and young adults with BPD symptoms. The VR-based approach offers advantages in experimental control and ecological validity, showing potential for both diagnostic assessment and therapeutic intervention in clinical settings.

## Introduction

High emotional arousal (EA) is a transdiagnostic phenomenon observed across several psychiatric disorders (Stiglmayr et al., 2008). It can be described as the experience of tension that is perceived as aversive, and is most prevalent and intense among patients with borderline personality disorder (BPD; Stiglmayr et al., 2005; Wolff et al., 2007). Psychosocial stressors such as feelings of failure, isolation, or rejection are primary triggers of EA. These stressors typically occur in clusters throughout daily life, causing rapid increases followed by a slow decrease in EA in patients with BPD (Stiglmayr et al., 2005).

Adolescent BPD patients particularly struggle with EA management as part of their broader emotion dysregulation (Ibraheim et al., 2017). Given that very high EA often results in long-term dysfunctional coping attempts such as non-suicidal self-injury (NSSI), it represents a crucial focus in BPD therapies such as dialectical behavior therapy (DBT; Chapman, 2019; Linehan & Wilks, 2015). A key therapeutic objective is to help patients develop new coping strategies in response to EA-triggering situations. However, simulating specific trigger situations during in-vivo therapy, especially sequential trigger scenarios that mirror patients' daily life experiences, remains challenging (Bush, 2008).

Virtual reality (VR) might have the potential to complement in-vivo therapy by creating a variety of scenarios that elicit EA. In recent years, VR has proven to be a valuable tool to enhance research and therapeutic methods by providing a highly structured, standardizable, and resource-efficient environment capable of simulating near-real-life situations (Rizzo et al., 2023). Due to these unique characteristics, VR has been shown to be especially effective in treating anxiety disorders and post-traumatic stress disorder (Horigome et al., 2020; Wiebe et al., 2022). In view of the promising findings regarding the treatment of emotion-based disorders, it might be assumed that VR could also offer significant benefits for individuals with BPD. However, research on VR applications for BPD remains limited. To date, only a small number of VR-based studies have included young adults or adolescents with BPD (Falconer et al., 2017; Kučerová, 2024; Liebke et al., 2018; McLachlan et al., 2022), and to the best of our knowledge, no study has specifically investigated EA in this population. These previous studies have primarily focused on other aspects of BPD symptomatology: understanding mental states (Falconer et al.), coping strategies to social stressors (Kučerová), beliefs about emotions in adolescents (McLachlan), and social acceptance dynamics (Liebke). While these investigations have established initial evidence for VR's feasibility with BPD populations across developmental stages, they have not directly addressed how EA—a core component of emotional dysregulation in BPD—can be systematically studied and potentially targeted through VR interventions.

In order to implement VR in diagnostic and therapeutic settings in a meaningful way, it is first essential to establish an experimental paradigm that is capable of reliably inducing EA in virtual environments. Such a paradigm must closely reflect the real-life experiences of young patients with BPD symptoms to determine whether EA can be elicited in VR at all. While several traditional stress paradigms have already been established in VR (Helminen et al., 2021; van Dammen et al., 2022), their use for this purpose faces several challenges. Many paradigms rely on a single type of stressor, such as social exclusion in the Cyberball paradigm (Kassner et al., 2012), which does not capture the complexity of real-life stressors experienced by BPD patients; some paradigms use triggers that are less relevant for adolescents and young adults, such as job interview scenarios in the Trier Social Stress Test (TSST; Zimmer et al., 2019), thus failing to reflect age-specific stress experiences; moreover, the scenarios are often not adapted to the social and emotional realities described by young BPD patients, such as rejection, isolation, or feelings of failure (e.g., virtual Stroop task; Poguntke et al., 2019). School environments represent particularly relevant and ecologically valid settings for adolescents and young adults, as they spend a significant portion of their time in these contexts and frequently encounter social stressors there, including peer evaluation, performance pressure, and social hierarchies (Núñez-Regueiro & Núñez-Regueiro, 2021). This also extends to school-like settings such as vocational schools, higher secondary education institutions, and universities, where similar social and academic demands persist and continue to shape socio-emotional development (Rončević & Marinić, 2024).

To address these limitations, we developed the VR-based *aVeRsive tension* paradigm, which represents a multi-stressor environment based on real-life social stressors described by adolescent and young adult patients with BPD symptoms. We hypothesized that all participants in our study would experience higher EA upon exposure to the socially induced multi-stressor setting in VR, and that EA would be significantly higher in young patients with BPD symptoms compared to healthy controls (HC). Furthermore, we examined the extent to which higher EA is reflected in psychophysiological measures.

## Methods

### Participants

All participants provided informed consent, and additional consent from legal guardians was obtained for participants under the age of 18. Participants received €120 compensation for taking part in the study.

To ensure participants’ safety, clear inclusion and exclusion criteria were applied. For inclusion in the BPD group, participants had to fulfil at least three diagnostic criteria for BPD according to the DSM-5 (American Psychiatric American Psychiatric Association, 2014). BPD patients with acute suicidality, acute psychotic disorders or unstable psychotropic medication, e.g. recent medication changes, were excluded. General exclusion criteria for both groups were binocular vision problems, stereo blindness, known hypersensitivity to cybersickness, epilepsy, and lack of informed consent.

The total sample consisted of 147 adolescents and young adults (*n* = 77 patients with BPD symptoms, *n* = 70 HC) aged between 13 and 25 years. Participants were recruited between February 2022 and January 2024 through local advertising and our psychiatric outpatient clinic at the University Hospital Wuerzburg as well as external facilities such as residential groups (*n* = 8) or psychiatric hospitals (*n* = 10). Although we initially preregistered a target sample size of *n* = 120 participants (see: https://aspredicted.org/gt9f-s6pb.pdf), further recruitment was necessary due to early dropout after providing consent but before data collection.

A total of 23 participants were excluded from the final analyses. In the BPD group, five individuals were excluded due to not meeting the diagnostic criteria after screening, and ten withdrew consent before completing data collection. In the HC group, two participants reported current or past psychiatric diagnoses during screening, one was excluded due to technical problems, and five withdrew consent prior to participation.

The final sample therefore comprised 62 participants in each group (Table 1). In the BPD group, 50 (80.65 %) participants were female, five (8.06 %) were male, and seven (11.29 %) were gender-diverse, and the mean age was 18.11 years (SD = 2.68). On average, participants in the BPD group fulfilled 5.42 BPD criteria (SD = 1.83), and 46 (74.19 %) were receiving ongoing psychotherapeutic treatment. A total of 39 patients (62.9 %) were taking at least one psychotropic drug. The most common drugs were antidepressants (50 %), neuroleptics (38.7 %), stimulants (12.9 %) and sleep medication (9.7 %). On average, the patients took 1.68 (± 1.12) psychotropic medications. In the HC group, 59 (95.16 %) participants were female, two (3.23 %) were male, and one (1.61 %) was gender-diverse, and the mean age was 18.21 years (SD = 2.73). None of the HC participants stated that they were taking psychotropic medication.

**Table 1:** Sample characteristics and group comparisons.

		BPD			HC			Group comparisons		
n		62			62					
		female	male	diverse	female	male	diverse	Fisher’s exact test		*P*
**gender n (%)**		50 (80.65 %)	5 (8.06 %)	7 (11.29 %)	59 (95.16 %)	2 (3.23 %)	1 (1.61 %)			**0.032**
			M	SD		M	SD	t	df	*P*
age in years			18.11	2.68		18.21	2.73	−0.199	122	0.842
BPD criteria			5.42	1.83						
current treatment n (%)		46 (74.19 %)			0					
medication										
at least one psychotropic medication n (%)		39 (62.9 %)			0					
antidepressants*		31 (50 %)								
*neuroleptics*		24 (38.7 %)								
*stimulants*		8 (12.9 %)								
*sleep medication*		6 (9.7 %)								
Patients with multiple medication classes		21 (33.9 %)								
								Welch *t*-test		
		n	M	SD	n	M	SD	t	df	*P*
**DSS**		59	3.55	2.49	61	0.38	0.96	9.157	74.479	**< 0.001**
**BSCL**		60	26.99	6.12	61	12.9	3.03	16.006	86.029	**< 0.001**
**SPAIC**		58	30.83	12.21	61	11.61	8.27	10.004	99.573	**< 0.001**
**NTS**										
*belonging*	**Stress**	53	2.14	0.82	57	2.74	0.98	−3.503	106.81	**< 0.001**
	**control**	54	4.04	1.23	57	5.3	0.77	−6.451	88.237	**< 0.001**
*control*	Stress	53	1.82	0.85	57	2.16	1.1	−1.831	104.39	0.070
	**control**	54	4.69	1.36	57	5.29	0.99	−2.625	96.741	**0.010**
*self*	**Stress**	53	3.15	1.58	57	4.48	1.21	−4.92	97.085	**<0.001**
	**control**	54	4.39	1.62	57	6.01	0.99	−6.343	86.733	**<0.001**
*meaning*	**Stress**	53	1.77	0.88	57	2.85	1.29	−5.139	99.368	**<0.001**
	**control**	54	3.59	1.49	57	4.98	1.03	−5.723	93.854	**<0.001**
**FMSQ**										
	**Stress**	55	5.51	4.58	60	2.5	2.37	4.365	79.337	**< 0.001**
	**control**	57	6.02	5.2	58	2.55	2.66	4.484	83.124	**< 0.001**
PRE										
	Stress	51	5.18	2.35	53	5.02	1.81	0.382	94.088	0.703
	control	53	5.38	2.47	53	4.87	1.83	1.205	95.771	0.231

*Note. M* = mean, SD = standard deviation, BPD = patients with borderline personality disorder symptoms, HC = healthy controls, BSCL = Brief Symptom Checklist, DSS = Dissociation Tension Scale acute, * Percentages sum to more than 100 % due to patients taking multiple medication classes, DSS-acute SPAIC = Social Phobia and Anxiety Inventory for Children, NTS = Need Threat Scale, FMSQ = Fast Motion Sickness Questionnaire, PRE = Presence.

### Design

The study used a quasi-experimental 2 (group: BPD/HC) x 2 (VR condition: stress/control) mixed between-within-subjects design, resulting in two VR trials per participant conducted on separate days, with a mean of 12.59 days (SD = 13.74) between the two trials. To account for logistical constraints, we employed a quasi-randomization approach to balance the order of trial days within groups, ensuring comparability across conditions. The VR trial in both the stress condition and the control condition comprised a modified Trier Social Stress Test (TSST; Kirschbaum et al., 1993) followed by a Cyberball paradigm (Williams et al., 2000). Since EA is reflected in both subjective and physiological measures, we also measured heart rate (HR), electrodermal activity (EDA), and salivary cortisol, which are well-established markers of stress-related physiological arousal. The study protocol was approved by the ethics committee of the University Hospital Wuerzburg (29/21-am).

### Procedure

During the initial appointment, participants were given detailed study information and provided informed consent. To confirm BPD symptoms in the patient group, the Structured Clinical Interview for DSM-5 personality disorders – borderline personality disorder section (SCID-5 PD; First et al., 2019) was administered by clinically trained staff. Following this appointment, participants completed online self-report questionnaires (see section 5. Measures).

**Fig. 1:**
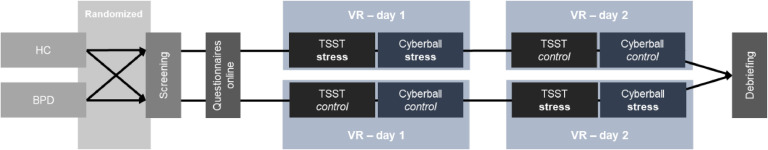
Study design. HC = healthy control participants, BPD = participants with borderline personality disorder symptoms, VR = virtual reality, TSST = Trier Social Stress Test.

Each VR trial began between 12:00 and 19:00. The sessions lasted for approximately 90 min, which included 25 min for setup and pretesting and another 25 min for dismantling the devices and the follow-up survey. The study was implemented according to a standardized protocol, as depicted in Fig. 1: After the study staff explained the VR apparatus to the participant and prepared the instruments, the VR session started. Each VR trial began with a preparation period, consisting of a nature setting (3 min) and a school setting (2 min) in order to acclimatize the participant to the VR and minimize the risk of novelty effects. Following this, participants completed an adapted TSST including a speech and mental arithmetic task (10 min) and the Cyberball paradigm (6 min). After completing the paradigms, participants had to answer some questions both within the VR (5 min; Need Threat Scale after social exclusion during Cyberball) and outside of the VR setting (10 min; questionnaires on VR experience). During the VR session, the virtual experimenter guided the participant through the experiment.

**Fig. 2:**
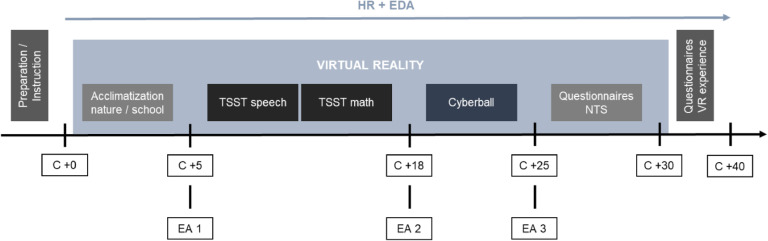
Experimental procedure and measures. HR = heart rate, EDA = electrodermal activity, TSST = Trier Social Stress Test, NTS = Need Threat Scale, *C* +minutes = cortisol measures at 6 time points, EA 1–3 = subjective emotional arousal rating.

The study included the following measures taken during each laboratory session: 1. Participants answered brief questions on subjective EA (EA 1–3). 2. Saliva samples were collected at six time points in line with the study protocol. 3. HR and EDA were recorded throughout the VR session (cf. Fig. 2). After the second VR trial, participants were fully debriefed about the aims of the study.

### Apparatus and virtual reality environment

The virtual reality environments were developed by the Department of Psychology of Intelligent Interactive Systems (Prof. Wienrich) at the University of Wuerzburg using Unity (version 2019.4.21f1). The application was run on a Razer Blade 15 gaming notebook with an Intel Core i7-10750H, a GeForce RTX 3060 and 16GB of RAM. We presented these environments through Oculus Rift S headsets with a field view of 110° (resolution of 1280 × 1440 pixels per eye with an 80 Hz refresh rate) and used touch controllers. We utilized realistic human 3D models as agents, with pre-recorded natural speech. During the speech and mental arithmetic tasks, the experimenter manually controlled the agent responses.

To maintain experimental control, participant movement was restricted to head motion and predetermined teleportation points. The environment integrated two sequential social stressor paradigms within a school-like setting, implementing both stress and control conditions.

The session began with an acclimatization phase, in which participants learned controller interactions in a natural setting before exploring the school environment (cf. Fig. 2), which included both a schoolyard and corridor.

#### Adaptation of the trier social stress test

**Fig. 3:**
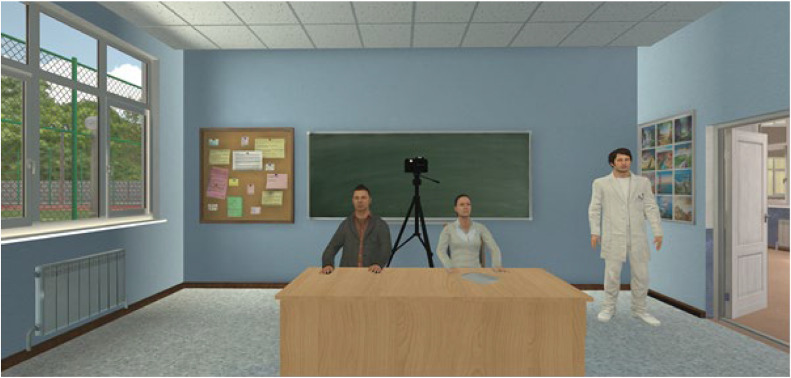
Trier Social Stress Test (TSST) in *aVeRsive tension*.

In an adaptation of a modified Trier Social Stress Test (TSST-M) by Yim et al. (2015), we asked participants to give a speech in school in front of two observer agents (TSSTs; one male and one female; see Fig. 3), instead of the original TSST protocol comprising a job interview. The task required participants to introduce themselves to a new class. After a one-minute preparation period, participants were required to present their speech for 5 min. If participants faltered, the experimenter could manually ask pre-recorded questions about the participant to keep the speech flowing. After the time expired, participants were required to complete the mental arithmetic task out loud for 5 min, which consisted of a sequential subtraction task (TSSTm).

The stress condition involved increased social evaluation pressure, which was realized through several mechanisms: At the beginning of the first VR trial, participants were given a cover story, which stated that the session might be video-recorded and might be observed by peers online. This was visually represented by a red blinking camera. Additionally, the observer agents showed less friendly facial expressions and emphasized the time remaining during the speech task. If participants made errors during the mental arithmetic task, the observers immediately demanded that the task be restarted.

In contrast, the control condition consisted of a friendly version of the TSST in line with Wiemers et al. (2013). Participants were informed that the observers were new and might make mistakes themselves. Moreover, the observer agents maintained friendly facial expressions throughout and did not request restarts after arithmetic errors.

#### Adaptation of the cyberball paradigm

Our Cyberball paradigm took place in the virtual schoolyard and lasted for a total of 6 min. As with the original Cyberball paradigm (Williams et al., 2000), we presented a VR-adapted ball-tossing game in which two computer-controlled agents and the participant throw a ball to each other (see Figure S3 in the supplementary material).

In the stress condition, the participant was excluded from the game after 33.3 % of the ball tosses, while the virtual agents threw the ball back and forth to each other for the remaining time. In the control condition, the participant received and threw the ball with the same frequency as the playing agents.

### Measures

#### Self-report data

Throughout the study, we assessed subjective emotional arousal (EA) based on the methodology of Stiglmayr et al. (2005). Participants rated their inner tension (‘How high was your inner tension right before the questioning?’) on a 10-point Likert scale (0–9) at three time points: before the TSST (EA 1), after the TSST (EA 2), and after the Cyberball paradigm (EA 3). Additionally, we collected comprehensive demographic data including age and gender, along with information about psychiatric diagnoses and current treatment status.

To assess participants’ tendency to dissociate, we used the Dissociation Tension Scale 4 acute (DSS-4; Stiglmayr et al., 2009), which encompasses four items on depersonalization, somatoform dissociation, derealization, and analgesia, rated on a 10-point Likert scale ranging from 0 (not present) to 9 (very strong). The DSS-4 has shown good internal consistency, with α = 0.87.

To evaluate general mental health differences between groups, we administered the Brief Symptom Checklist (BSCL; Franke, 2017). This 53-item questionnaire examines nine dimensions of psychopathology: aggressiveness/hostility, anxiety, depression, paranoid thinking, phobic anxiety, psychoticism, somatization, social insecurity, and compulsiveness. Participants rated their symptoms on a 5-point Likert scale ranging from 0 (not at all) to 4 (very strong). The BSCL has shown varying internal consistencies across the different subscales, ranging from α = 0.39 for phobic anxiety to α = 0.72 for both depression and compulsiveness (Franke, 1997).

To control for influences of social phobia symptoms on subjective emotional arousal in socially induced stress situations, we used a German version of the Social Phobia and Anxiety Inventory for Children (SPAIC; Beidel, 1996). The 26 items were rated on a 3-point Likert scale from 1 (never or rarely) to 3 (mostly or always). The questionnaire has shown good internal consistency, with α = 0.92 (Melfsen et al., 2011).

For a manipulation check, we assessed the effects of social inclusion and exclusion after Cyberball using the Need Threat Scale (NTS) in line with Van Beest and Williams (2006). The NTS includes the subscales ‘belongingness’, ‘self-esteem’, ‘control’, and ‘meaningful experience’, each containing five items, which are rated on a 7-point Likert scale ranging from 1 (do not agree) to 7 (agree). The scale is widely recognized as a standard measure in Cyberball research (Hartgerink et al., 2015).

To confirm the tolerability of the VR application, we measured simulator sickness after the experiment using the Fast Motion Sickness Questionnaire (FMSQ; Keshavarz & Hecht, 2011), with participants rating the extent of nausea they experienced during participation on a 20-point scale. Additionally, the sense of presence (PRE) was assessed using a single item: ‘To what extent did you feel present in the virtual environment, as if you were really there?’, rated on a Likert scale ranging from 1 (very little) to 10 (very strong; Bouchard et al., 2004).

#### Physiological measures

To continuously monitor physiological stress responses throughout the VR trials, we used the Empatica E4 wristband (Empatica Inc.). The device was worn on the wrist of participants’ dominant hand and recorded HR at 1 Hz, capturing stress-induced sympathetic activity from the beginning of the experiment until the completion of the final interview. To assess sympathetic activity, we also used the Empatica E4 to measure EDA. For the assessment of endocrine stress responses, we collected saliva samples to measure salivary cortisol.

Since HR is a continuous measure with a higher temporal resolution than cortisol, and wrist measurement of HR is better validated than EDA (Milstein & Gordon, 2020), we will primarily report HR data. Detailed information on EDA and cortisol is provided in the supplementary material (S1).

### Data analysis

#### Data curation of physiological measures

The Empatica E4 software provided the average HR in spans of 10 s, extracted from the blood volume pulse (BVP) signal with a sampling rate of 1 Hz (Milstein & Gordon, 2020). We defined specific regions of interest (ROIs; baseline, TSSTs, TSSTm, Cyberball) in accordance with the log data of the experimental VR file and removed outlier values that deviated > 3 SD from the group mean per condition and ROI (*n* = 9481/729,619 observations).

For data curation of EDA measures and cortisol, please see the supplementary material (S2).

#### Statistical analyses

Data analysis was conducted using R (version 4.3.3). For demographic comparisons, we analyzed gender differences between the BPD and HC group using Fisher's exact test, and assessed age differences using independent samples *t*-tests. Group differences in dissociation tendency (DSS), psychopathology (BSCL), social anxiety (SPAIC), and need threat (NTS) as well as simulator sickness (FMSQ) and presence (PRE) were evaluated using Welch’s *t*-tests to account for inequality of variance.

In the preregistration of the study, we stated that we would exclude participants with missing EA ratings in VR (https://aspredicted.org/gt9f-s6pb.pdf). However, due to unexpectedly high rates of missing data, mainly caused by technical problems during data collection, we deviated from the original analysis plan. The missing data appeared to occur at random and did not differ systematically between groups. Therefore, we opted to use linear mixed models (LMM) using the 'lmerTest' package (Kuznetsova et al., 2017), which can handle missing data more efficiently while maintaining statistical power. This approach allowed us to include partial data from participants who completed at least one laboratory day or had occasional missing ratings.

To test whether the TSST successfully manipulated subjective EA, we calculated an LMM with the fixed effects group (BPD/HC), condition (stress/control) and ROI (baseline/EA 1). In order to control for possible habituation effects over the course of the study, we integrated the study day (1/2) as a factor. Furthermore, we tested for interactions between group, condition and ROI, and included random intercepts for participants to account for repeated measures. As a manipulation check for the Cyberball paradigm, we calculated four LMMs for each subscale of the NTS (belonging, self, control, meaning) in the HC group, with condition (stress/control) and study day (1/2) as fixed effects and participants as a random effect.

For our primary analyses, we developed two models, a simple and a more complex model, for each dependent variable (subjective EA, HR, cortisol, EDA). The initial models included the main predictors (ROI, condition, group, study day) and basic interactions (ROI x condition x group), while the complex models incorporated z-standardized covariates (age, social anxiety, dissociation tendency). Participant was included as a random intercept.

Model selection relied on Akaike information criterion (AIC) and Bayesian information criterion (BIC) comparisons using ANOVAs. The models were fitted using maximum likelihood estimation, and *p*-values were calculated using Satterthwaite's method for degrees of freedom, maintaining a significance threshold of *p* < 0.05.

Finally, we performed pairwise two-tailed *t*-tests to examine the pattern of EA between TSST (EA 2) and Cyberball (EA 3) for each group x condition dyad (four *t*-tests) separately. The alpha level was Bonferroni-adjusted to 0.0125 for these analyses (i.e., 0.05 divided by 4).

## Results

### Descriptive analyses

Table 1 presents descriptive data and group comparisons for sociodemographic variables and manipulation check measures. The BPD group comprised more participants that identify as gender-diverse (*p* = 0.032) and showed higher levels of dissociation tendency (*t*(74.479) = 9.157, *p* < 0.001), psychopathology (*t*(86.029) = 16.006, *p* < 0.001), and social anxiety (*t*(99.573) = 10.004, *p* < 0.001) than the HC group. On average, the BPD group fulfilled 5.42 (SD = 1.83) diagnostic criteria for BPD, with 74.19 % of the patients undergoing treatment at the time of study participation. Across both conditions, the groups differed significantly on all NTS subscales (all *p* < 0.01), with the exception of the control subscale in the stress condition. Overall, the BPD group reported lower feelings of belonging, control, self-esteem, and meaningful existence compared to the HC group. While the BPD group reported stronger simulator sickness in both conditions (*p* < 0.001), the groups did not differ regarding the experience of presence in VR.

A detailed analysis of manipulation checks of the TSST and Cyberball is provided in the supplementary materials (S3.1).

### Main analyses

#### Subjective ratings of emotional arousal (EA)

The average scores for subjective EA are displayed in Table 2. The LMM model comparison revealed that the more complex model with additional covariates fitted the data significantly better than the simpler model (χ²(3) = 37.894, *p* < 0.001). This was also reflected in the lower AIC and BIC values of the more complex model (AIC = 2588.7, BIC = 2670.4 vs. AIC = 2620.6, BIC = 2688.7 for the simpler model). The results for the complex LMM of subjective EA are presented in detail in Table 3.

**Table 2:** Mean outcome measures of subjective EA for the BPD and HC groups at all ROIs.

		BPD		HC	
ROI	condition	N	M (SD)	n	M (SD)
EA 1	control	61	4.43 (1.81)	64	2.22 (1.70)
EA 1	stress	57	4.33 (1.86)	65	2.20 (1.48)
EA 2	control	61	6.16 (2.09)	64	3.11 (2.18)
EA 2	stress	57	6.02 (2.12)	65	3.62 (2.07)
EA 3	control	59	4.90 (2.16)	61	1.79 (1.03)
EA 3	stress	56	5.29 (2.13)	62	2.50 (1.56)

*Note.* ROI = region of interest, EA 1–3 = subjective emotional arousal at time points 1–3, BPD = patients with borderline personality disorder symptoms, HC = healthy controls.

**Table 3:** Linear mixed model of subjective EA.

Predictors	Estimates	CI	*P*
**(Intercept)**	**5.37**	**4.83 – 5.90**	**<0.001**
**ROI [EA 2]**	**1.76**	**1.26 – 2.25**	**<0.001**
ROI [EA 3]	0.49	−0.01 – 0.99	0.054
condition [STRESS]	−0.11	−0.62 – 0.40	0.682
**group [HC]**	**−2.26**	**−2.88 – −1.64**	**<0.001**
zage	0.04	−0.17 – 0.26	0.692
**zSPAIC**	**0.49**	**0.27 – 0.72**	**<0.001**
**zDSS**	**0.39**	**0.16 – 0.62**	**0.001**
**study day**	**−0.62**	**−0.83 – −0.42**	**<0.001**
ROI [EA 2] × condition [STRESS]	−0.04	−0.76 – 0.68	0.909
ROI [EA 3] × condition [STRESS]	0.51	−0.21 – 1.23	0.168
**ROI [EA 2] × group [HC]**	**−0.79**	**−1.48 – −0.10**	**0.025**
**ROI [EA 3] × group [HC]**	**−0.92**	**−1.62 – −0.22**	**0.010**
condition [STRESS] × group [HC]	0.11	−0.59 – 0.81	0.757
(ROI [EA 2] × condition [STRESS]) × group [HC]	0.51	−0.48 – 1.50	0.312
(ROI [EA 3] × condition [STRESS]) × group [HC]	0.22	−0.78 – 1.22	0.668
random effects			
σ^2^	1.85		
τ00 VP	1.09		
ICC	0.37		
N VP	119		
observations	693		
marginal R^2^ / conditional R^2^	0.493 / 0.680		

*Note.* CI = confidence interval, ROI = region of interest, EA 1–3 = subjective emotional arousal at time points 1–3, with EA 1 serving as the reference category, HC = healthy controls, zSPAIC = *z*-standardized values of Social Phobia and Anxiety Inventory for Children, zDSS = *z*-standardized scores of dissociation tendency, ICC = intraclass correlation coefficient.

The analysis revealed significant main effects of group (*b* = −2.26, *p* < 0.001) in the assessment after the TSST (EA 2: *b* = 1.76, *p* < 0.001). HC and patients differed in terms of their pattern of EA, as shown by the significant interactions between group and two ROIs (EA 2 × HC: *b* = −0.79, *p* = 0.025; EA 3 × HC: *b* = −0.92, *p* = 0.010). Pairwise comparisons revealed distinct patterns of EA between groups and conditions: In the control condition, both the BPD and HC group showed significant decreases in EA from TSST to Cyberball (*p* ≤ 0.001). However, in the stress condition, only the HC group showed a significant decrease in EA (*p* < 0.001), while the BPD group maintained elevated EA levels (*p* = 0.232; see Table 4).

**Table 4:** Pairwise comparisons of EA 2 and EA 3 per group and condition.

contrast		estimate	SE	Df	z-ratio	*Bonferroni adj. p*
**Control BPD EA 2**	**Control BPD EA 3**	**1.266**	**0.255**	**564**	**4.962**	**< 0.001**
Stress BPD EA 2	Stress BPD EA 3	0.716	0.266	564	2.694	0.232
**Control HC EA 2**	**Control HC EA 3**	**1.393**	**0.250**	**564**	**5.575**	**< 0.001**
**Stress HC EA 2**	**Stress HC EA 3**	**1.134**	**0.248**	**564**	**4.574**	**< 0.001**

BPD = patients with borderline personality disorder symptoms, HC = healthy controls, SE = standard error, df = degrees of freedom, Bonferroni-adjusted *p*-value = 0.0125.

Higher levels of social anxiety (β = 0.49, *p* < 0.001) and higher tendencies to dissociate (β = 0.39, *p* = 0.001) predicted higher subjective EA scores. The EA scores decreased over the two study days (*b* = −0.62, *p* < 0.001). There were no significant effects of experimental condition and age, and no three-way interaction (all *p*s > 0.05).

**Fig. 4:**
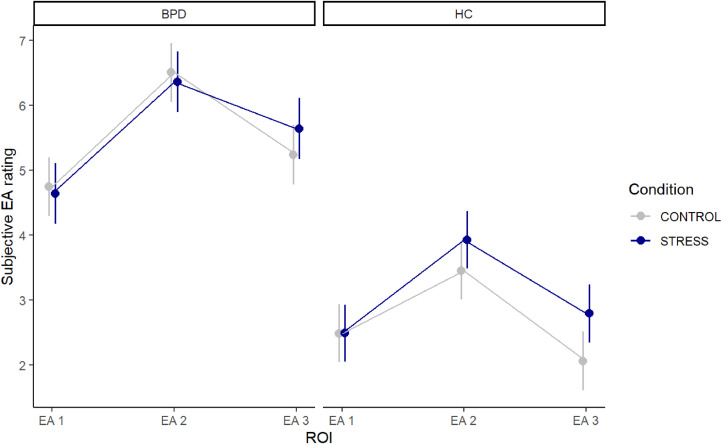
Predicted values of the linear mixed model of EA. *Note.* EA 1–3 = emotional arousal at (1) baseline, (2) after TSST, and (3) after Cyberball, BPD = patients with borderline personality disorder symptoms, HC = healthy controls, ROI = region of interest.

Fig. 4 shows the predicted values of subjective EA for both groups across the three ROIs and both conditions.

#### Physiological data: heart rate (HR)

Table 5 presents the average scores for the HR measures. Although the more complex model with additional covariates showed a higher BIC and minimal improvement (χ²(3) = 10.936, *p* = 0.012) compared to the simpler model, the simpler model was chosen for reasons of parsimony. The results for the simpler LMM of HR measures are presented in detail in Table 6.

**Table 5:** Mean heart rate [Hz] and standard deviation of BPD and HC group for each ROI.

		BPD		HC	
ROI	condition	n	M (SD)	n	M (SD)
Acc	control	54	90.13 (15.60)	50	84.53 (12.36)
Acc	stress	44	87.97 (14.05)	48	85.61 (12.76)
TSSTs	control	55	92.84 (17.51)	51	87.24 (13.95)
TSSTs	stress	48	94.49 (15.87)	53	89.22 (14.61)
TSSTm	control	55	90.63 (17.90)	51	86.88 (15.87)
TSSTm	stress	47	91.02 (16.48)	53	89.65 (19.22)
Cyberball	control	55	90.51 (16.64)	50	89.00 (14.87)
Cyberball	stress	49	92.44 (17.04)	53	86.55 (13.80)

*Note.* HR = heart rate, ROI = region of interest, Acc = Acclimatization, TSSTs = Trier Social Stress Test speech task, TSSTm = Trier Social Stress Test mental arithmetic task, BPD = patients with borderline personality disorder symptoms, HC = healthy controls, *M* = mean, SD = standard deviation.

**Table 6:** Linear mixed model of HR.

Predictors	Estimates	CI	*P*
**(Intercept)**	**89.72**	**88.06 – 91.38**	**<0.001**
**ROI [TSSTs]**	**3.66**	**3.35 – 3.97**	**<0.001**
**ROI [TSSTm]**	**1.48**	**1.17 – 1.79**	**<0.001**
**ROI [Cyberball]**	**1.38**	**1.07 – 1.69**	**<0.001**
**condition [STRESS]**	**−2.51**	**−2.86 – −2.17**	**<0.001**
**group [HC]**	**−4.17**	**−5.26 – −3.07**	**<0.001**
**study day**	**−0.24**	**−0.35 – −0.12**	**<0.001**
**ROI [TSSTs] × condition [STRESS]**	**4.03**	**3.56 – 4.49**	**<0.001**
**ROI [TSSTm] × condition [STRESS]**	**2.89**	**2.43 – 3.35**	**<0.001**
**ROI [Cyberball] × condition [STRESS]**	**4.04**	**3.60 – 4.49**	**<0.001**
**ROI [TSSTs] × group [HC]**	**−1.64**	**−2.10 – −1.18**	**<0.001**
ROI [TSSTm] × group [HC]	0.19	−0.27 – 0.65	0.417
**ROI [Cyberball] × group [HC]**	**2.02**	**1.57 – 2.47**	**<0.001**
**condition [STRESS] × group [HC]**	**3.33**	**2.84 – 3.82**	**<0.001**
**(ROI [TSSTs] × condition [STRESS]) × group [HC]**	**−2.46**	**−3.11 – −1.80**	**<0.001**
(ROI [TSSTm] × condition [STRESS]) × group [HC]	−0.57	−1.22 – 0.09	0.090
**(ROI [Cyberball] × condition [STRESS]) × group [HC]**	**−6.48**	**−7.11 – −5.84**	**<0.001**
random effects			
σ2	185.45		
τ00 VP	70.84		
ICC	0.28		
N VP	113		
observations	240,506		
marginal R2 / conditional R2	0.024 / 0.294		

*Note.* HR = heart rate, ROI = region of interest, HR = healthy controls, TSSTs = Trier Social Stress Test speech task, TSSTm = Trier Social Stress Test mental arithmetic task, ICC = intraclass correlation coefficient, CI = confidence interval.

The model of HR measures showed significant three-way interactions between ROI, condition, and group for the ROIs *TSSTs* (*b* = −2.46, *p* < 0.001) and *Cyberball* (*b* = −6.48, *p* < 0.001). These interactions show that stress reactivity manifested differently between the groups depending on the condition: While both groups showed an increase in HR under stress (*b* = −2.51, *p* < 0.001), this increase was more pronounced in the BPD group, particularly during the *Cyberball* paradigm (cf. Table 5).

There was a significant main effect of HR over the two study days (*b* = −0.24, *p* < 0.001), with HR decreasing over time in all participants.

**Fig. 5:**
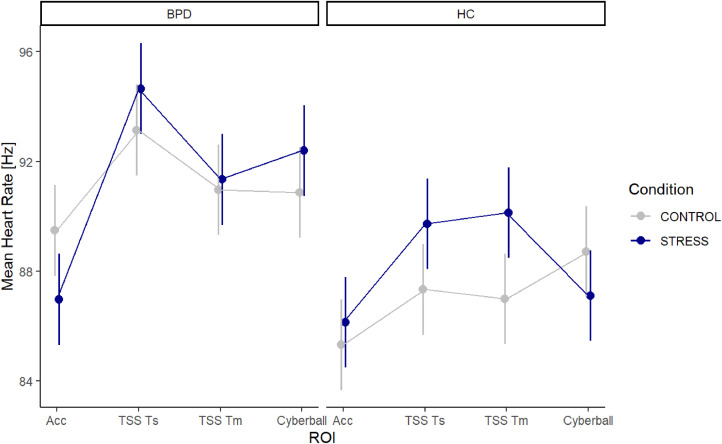
Predicted values of the linear mixed model of heart rate [Hz]. *Note.* Acc = Acclimatization, TSSTs/*m* = Trier Social Stress Test speech task/mental arithmetic task, BPD = patients with borderline personality disorder symptoms, HC = healthy controls, ROI = region of interest.

Fig. 5 presents the predicted values of HR measures for both groups across the four ROIs and both conditions.

## Discussion

The present study successfully validated the novel *aVeRsive tension* paradigm, designed to assess EA in a VR setting among adolescents and young adults with BPD symptoms. Using two established social stress paradigms (TSST, Kirschbaum et al., 1993; Cyberball, Williams et al., 2000) in a controlled VR environment, we demonstrated that *aVeRsive tension* allows for effective discrimination between patients with BPD symptoms and HC, capturing both subjective and physiological responses. The findings provide robust evidence that VR can simulate real-life stressors, thus offering a valuable tool for understanding the dynamics of EA in individuals with BPD symptoms.

The findings support our hypothesis of heightened EA following socially stressful situations in our *aVeRsive tension* paradigm: The two groups showed a similar pattern of EA during the experiment, with peak levels observed after the TSST. However, patients with BPD symptoms consistently showed more pronounced peaks compared to HC participants, suggesting that BPD patients experience more intense emotional reactions. Contrary to our expectation, the two conditions (stress vs. control) did not elicit differing EA responses in either group, suggesting that the socially induced stress condition may have been insufficient to produce persistent, distinct EA responses across conditions. Moreover, we observed differences in the development of EA from the TSST to Cyberball: The BPD group showed a significant decrease in EA in the control condition but no significant decrease in the stress condition, whereas the HC group showed a significant decrease in EA in both conditions.

The HR data support the subjective ratings, with the BPD group showing consistently higher HR levels compared to the HC group, suggesting altered autonomic arousal in patients with BPD symptoms. The partially elevated HR values in the stress condition indicate that the differences in subjective EA between the conditions were reflected at least to some extent in physiological markers, although these differences were not consistently observed across all tasks. Both the subjective ratings and HR were significantly influenced by study day, which may indicate habituation effects over time.

While the EDA data showed an increasing trend, cortisol levels decreased over the course of the experiment. Given the limited validity of EDA measurement (Milstein & Gordon, 2020) and the methodological constraints of cortisol measures (see *Limitations*), we refrain from drawing definitive conclusions here. The divergent findings of EDA and salivary cortisol are presented in the supplement for the sake of completeness (S3). Future research may explore whether these patterns reflect distinct physiological aspects of EA.

Previous research examining the combined paradigms of TSST and Cyberball is limited (Weik et al., 2010, 2013; Williamson et al., 2018), with only one previous study conducted in VR (Kothgassner, Goreis, Glenk, Kafka, Beutl et al., 2021, Kothgassner, Goreis, Glenk, Kafka, Pfeffer, et al., 2021). As such, our findings contribute novel insights into EA responses across different social contexts in patients with BPD symptoms.

The observed increase in EA and HR during the adapted TSST is in line with previous research, both in HC (adults: Man et al., 2023; adolescents: Seddon et al., 2020) and individuals with BPD (Deckers et al., 2015; Wingenfeld et al., 2018). In view of the characteristic of heightened sensitivity to rejection in individuals with BPD (Cavicchioli & Maffei, 2020; Látalová et al., 2023), we hypothesized that social exclusion would trigger elevated EA (Gratz et al., 2013) and possibly amplify the prior stress response (cf. Stiglmayr et al., 2005). However, our results revealed a different pattern: An intense EA response exclusively occurred after the TSST, and was followed by a relaxation phase during the Cyberball paradigm, characterized by decreased EA. This finding underlines the importance of task-specific factors in understanding EA responses in BPD.

Two conceivable explanations for our findings can be suggested. First, in line with previous research, the TSST may elicit stronger stress responses compared to other paradigms (Sequeira et al., 2021), resulting in a spillover effect of EA that overrides potential EA responses elicited by Cyberball. Moreover, the observed pattern may reflect a prolonged latency in EA reduction in the BPD group, which reflects a core characteristic of emotion dysregulation in BPD (Bortolla et al., 2019).

Second, the Cyberball paradigm may not have generated sufficient EA in patients with BPD symptoms in our VR setting. Previous research has yielded mixed findings regarding EA responses induced by Cyberball: While some studies found heightened EA elicited by Cyberball (Chapman et al., 2014; Gratz et al., 2013), others did not (Jobst et al., 2014; Seidl et al., 2020). To the best of our knowledge, the present study is the first VR-based study to employ the Cyberball paradigm in patients with BPD symptoms, underscoring the need for future studies to disentangle task-specific and condition-specific effects on EA by systematically varying the paradigms and their sequence.

In addition to the task-specific characteristics, it is illuminating to briefly discuss further findings observed in the present study: First, unlike previous research (Wiemers et al., 2013; Yim et al., 2015), we found no differences in subjective ratings between conditions when applying a more friendly version of the TSST as a control condition. We posit that the conditions may not have been sufficiently distinct to elicit different subjective experiences. Second, potential habituation effects across repeated exposure to similar social stress scenarios (Kudielka et al., 2007) might have attenuated participants’ subjective and physiological responses on the second study day. This habituation phenomenon is supported by recent VR based findings from Kothgassner et al. (2021), who demonstrated significant response diminution when administering the TSST at similar intervals of approximately one week. Their research showed that by repeated exposure, stress markers had habituated considerably, with both physiological (cortisol, heart rate) and subjective responses becoming significantly reduced compared to initial exposures. In our study, similar mechanisms likely affected stress reactivity, as participants may have developed anticipatory cognitive frameworks that moderated subsequent responses. Future studies should therefore incorporate more established control conditions (e.g., Wu et al., 2019) in order to better differentiate between experimental and control scenarios, while controlling for potential habituation effects.

The VR quality assessment revealed acceptable presence ratings which are similar to those reported in other VR studies involving adolescents (Buerger & Wienrich, 2024), further underscoring the ecological validity and applicability of the paradigm. Notably, patients with BPD symptoms reported higher levels of simulator sickness compared to HC participants, although this discrepancy may reflect heightened arousal and an overestimation of physiological states characteristic of the disorder (cf. Bortolla et al., 2020). Future research is necessary to investigate this phenomenon in more detail, including potential interactions between emotion dysregulation and perceived VR side effects.

### Limitations and strengths

Several limitations of the present study should be mentioned. First, the inconsistent results across physiological markers may be partly attributable to the fact that we did not measure hormonal influences such as the menstrual cycle phase and the use of hormonal contraceptives, which can affect cortisol responses (Narvaez Linares et al., 2020). Additionally, while EDA increased as a trend, its limited reliability in mobile measurements and potential movement artifacts (Milstein & Gordon, 2020) impede the ability to interpret these findings.

Second, while our 2 × 2 design captures the combined effects of the TSST and Cyberball paradigms, it does not allow for conclusions about the unique contributions of each paradigm to EA responses. Specifically, it remains unclear what level of EA participants would have shown had we employed the Cyberball paradigm alone. Furthermore, we did not explore whether a different sequence of paradigms—such as Cyberball followed by TSST— might have influenced combined EA. Future studies could address these questions by using a 4 × 4 design evaluating each paradigm individually as well as in various combinations. This approach would require a significantly larger sample size, which was beyond the scope of the present study.

While our predominantly female BPD group reflects the typical gender distribution observed in clinical settings for this disorder (approximately 75 % female; American Psychiatric American Psychiatric Association, 2014; Bozzatello et al., 2024), the lack of gender-diverse participants in the HC group limits our ability to draw conclusions about gender-specific patterns in EA. Moreover, the relevance of school-related stressors may have been lower for the older participants, given that compulsory education in Germany ends at age 15/16 years. Furthermore, the focus on a BPD sample prevents us from drawing broader conclusions about EA in patients with different psychiatric disorders. These limitations underline the need for further research to refine both the methodological design and practical applications of the *aVeRsive tension* protocol. Future investigations should further aim to enhance the adaptability of the protocol across diverse populations and settings, to ensure its robustness and utility for both research and clinical practice.

It is important to note that two participants experienced dissociative episodes, indicating extreme EA levels beyond their regulatory capacity. Both patients successfully applied DBT skills under clinical supervision, emphasizing the need for the following safety measures when applying VR stress protocols. These include mandatory clinical supervision during protocol administration, comprehensive pre-procedure briefing on potentially intense emotional responses, and immediate access to trained clinicians familiar with DBT interventions. These safeguards are essential for maintaining patient safety while preserving the protocol’s therapeutic value. Additionally, the successful resolution of dissociation through DBT validates the integration of therapeutic techniques into the protocol framework.

The present study is also characterized by several strengths. First, the study provides a comprehensive multimodal database on EA in patients with BPD symptoms during adolescence and young adulthood (13–25 years), a critical period for the onset and manifestation of symptoms. This is particularly relevant for social situations during adolescence and the associated symptom complexes of BPD. Second, the applied paradigms were well-validated and were specifically designed to address socially relevant triggers such as evaluation anxiety, rejection, and exclusion. As such, they are highly valuable for applications in emotional exposure therapy within the DBT framework for BPD, particularly for adolescent patients.

Third, the *aVeRsive tension* protocol is one of only a small number of paradigms developed specifically for VR environments to simulate real-life social stressors for adolescents, making it a methodologically innovative approach.

Fourth, the successful resolution of dissociative episodes using DBT strategies highlights the safety and therapeutic integration of the protocol. Finally, the focus on an underrepresented group—adolescents and young adults with BPD symptoms—fills a gap in the research and provides critical insights for this developmental phase.

### Implications

Our research establishes a foundation for VR applications in both preventive care and clinical intervention for patients with BPD symptoms.

The developed multi-stressor paradigm effectively induces EA in adolescent and young adult patients in a resource-efficient manner. Moreover, the *aVeRsive tension* protocol can be administered independently of location, thus offering significant advantages for clinical practice. By utilizing VR technology, therapists can guide patients through social scenarios that closely resemble real-life situations, providing support and targeted interventions during emotionally challenging moments. Patients in the present study exhibited EA peaks averaging 6.02 (SD = 2.12; Table 2) on a 0–9 scale—a level sufficient to trigger emotional responses without inducing the dissociative states commonly observed during extreme tension in BPD patients. This controlled level of EA suggests an optimal therapeutic window for EA-focused interventions, aligning with the principles of DBT (Kaess & Brunner, 2016). Moreover, simulating everyday stressors in this way bridges the gap between therapy sessions and real-world applications, potentially increasing the effectiveness of DBT by fostering a more direct transfer of skills from the therapeutic setting to daily life. Compared to traditional therapy rooms, VR-based scenarios create a highly valid and controlled environment for emotional exposure, allowing therapists to address specific triggers with greater precision.

The self-report measures provided strong evidence for the paradigm’s validity, reinforcing self-report measures as the most reliable clinical assessment tools (Rosenthal et al., 2008). Although physiological measurements showed greater variability, their continued use is recommended to establish more robust physiological markers of EA for clinical practice.

Future clinical applications may benefit from additional refinements to the stress paradigm. Key areas for investigation include modifying the temporal spacing between paradigm components, and incorporating patient feedback on stressor intensity to better align with symptom severity and treatment goals. Moreover, it might be beneficial to restructure the stressor sequence to avoid placing the strongest stimulus first, thereby minimizing potential carryover effects. Building on previous research (Kothgassner, Goreis, Glenk, Kafka, Beutl et al., 2021, Kothgassner, Goreis, Glenk, Kafka, Pfeffer, et al., 2021), future studies should examine how different sequences of *aVeRsive tension* components affect EA outcomes.

### Conclusion

Our controlled study validates the *aVeRsive tension* protocol, which offers several advantages over traditional stress tests. This approach is uniquely valuable through its incorporation of multiple stress situations that mirror patients' real-world experiences, its targeted focus on the developmental period when BPD symptoms typically emerge and crystallize, and its integration of diverse social stressors aligned with patients' emotional experiences.

The use of VR in psychotherapy for adolescents and young adults appears promising. On the one hand, VR resonates particularly well with younger populations, offering the potential to increase commitment and treatment adherence for both therapists and patients (Flujas-Contreras et al., 2020). By providing a clearly structured environment, VR enables direct work on specific issues with a practical connection to everyday life. The VR-based design of *aVeRsive tension* balances experimental control with ecological validity, making it especially suitable for adolescent and young adult populations.

The paradigm may be beneficial for both diagnostic assessment and therapeutic intervention. The consistent self-report measures provided strong validation of the protocol's effectiveness, while the successful management of dissociative episodes confirmed its clinical safety when properly implemented. Further research into physiological responses during *aVeRsive tension* exposure could deepen our understanding of patterns of EA in adolescents and young adults presenting with BPD symptoms, potentially leading to more targeted and effective interventions within the DBT framework.

## Funding

The Kaufmaennische Krankenkasse (KKH) funded the study. Contact address: KKH (Kaufmaennische Krankenkasse), Prevention and self-help department, Tobias Bansen, Karl-Wiechert-Allee 61, 30625 Hannover, Germany. None of the funding partners had a role in the design and conduct of the study or the writing of the manuscript. The KKH does not play a role in the data collection, analysis, interpretation of the data, and writing of publications. This publication was supported by the Open Access Publication Fund of the University of Wuerzburg.

## Declaration of competing interest

Nothing to declare.

## Appendix Supplementary materials

Image, application 1

Image, application 2
