# Supplementary material for: aVeRsive tension: A new virtual reality paradigm to assess emotional arousal in adolescent and young adult patients with symptoms of borderline personality disorder
Source: Int J Clin Health Psychol. 2025 May 5;25(2):100583. doi: 10.1016/j.ijchp.2025.100583 (PMC12138408; doi:10.1016/j.ijchp.2025.100583)
Supplement: Supplementary file 1 [file mmc1.docx]

Supplementary Material

S1. Physiological measures

*Electrodermal activity (EDA)*

Comparable to the HR assessment, we measured EDA at 4Hz using an Empatica E4 wristband worn on the wrist of the participant’s dominant hand.

*Cortisol*

To assess the endocrine stress response, participants provided saliva samples using Salivettes (Salivette®, Sarstedt, Wiener Neudorf, Austria) for the measurement of salivary cortisol. We developed a novel collection procedure that maintained immersion in the virtual environment. Participants approached a designated table in the virtual school corridor, where they could temporarily switch their VR display mode to view the real laboratory environment in a blurred state. This allowed them to collect their samples independently, with study staff providing timing guidance. After 60 seconds, participants returned to full VR immersion. Saliva samples were collected at six time points per session: before starting (region of interest, ROI: prestart; C +0 minutes), after acclimatization (ROI: acclimatization; C +5 minutes), following the TSST (ROI: TSST; C +18 minutes), after Cyberball (ROI: Cyberball; C +25 minutes), upon completing the VR questionnaires (ROI: VRQuestionnaires; C +30 minutes), and after the final non-VR assessments (ROI: post; C +40 minutes). Individual sampling times varied slightly (±3 minutes) due to variations in questionnaire completion rates between the saliva collections. The saliva samples were analyzed at the laboratory of Clemens Kirschbaum (Technische Universitaet Dresden). Samples remained frozen at -20 degrees Celsius until analysis, whereupon they were thawed and centrifuged at 3,000 rpm for 5 minutes to obtain clear supernatant. Salivary cortisol concentrations were measured using a commercially available chemiluminescence immunoassay with high sensitivity (IBL International, Hamburg, Germany). The intra- and interassay coefficients of variation for cortisol were below 9%, respectively.

S2. Data curation

*EDA*

The Empatica E4 provided EDA measurements ranged from 0.01 to 100 µS at a 4 Hz sampling rate. For the pre-processing of EDA measures, we used Matlab (version 8.11) in combination with ledalab (Benedek & Kaernbach, 2010; version 3.4.9). We applied a first-order Butterworth low-pass filter with 1Hz cutoff to the raw signal. We extracted the individual global mean [µS] for each ROI (baseline, TSSTs, TSSTm, Cyberball) per condition, removing outliers using the same three standard deviation criterion (n = 96/1062 observations).

*Cortisol*

For cortisol analysis, we calculated average levels from laboratory duplicates for each measurement time point (ROIs 1-6). Following the same protocol as the HR data, we removed outliers beyond three standard deviations from the group mean per condition and region (n = 101/1338 observations).

S3. Results

S3.1 Manipulation checks

The analysis of the TSST manipulation check (Table 1S) revealed that all participants showed a significant increase in subjective EA immediately after the TSST (b = 1.74, *p* < 0.001), although this was less pronounced in the HC than in the BPD group (group × ROI: b = -0.85, *p* = 0.015). There was also a significant influence of the study day (b = -0.66, *p* < 0.001). We found no significant effects of condition and no interaction effects (all *p*s > 0.05).

Table S1. Linear mixed model of subjective EA for TSST manipulation check.

| Predictors | Estimates | CI | *p* |
| --- | --- | --- | --- |
| **(Intercept)** | **5.38** | **4.78 – 5.97** | **<0.001** |
| **group [HC]** | **-2.16** | **-2.82 – -1.49** | **<0.001** |
| condition [STRESS] | -0.06 | -0.57 – 0.44 | 0.804 |
| **ROI [EA 2]** | **1.74** | **1.25 – 2.23** | **<0.001** |
| **study day** | **-0.66** | **-0.90 – -0.41** | **<0.001** |
| group [HC] × condition [STRESS] | 0.02 | -0.67 – 0.71 | 0.953 |
| **group [HC] × ROI [EA 2]** | **-0.85** | **-1.53 – -0.17** | **0.015** |
| condition [STRESS] × ROI [EA 2] | -0.05 | -0.75 – 0.65 | 0.881 |
| (group [HC] × condition [STRESS]) × ROI [EA 2] | 0.58 | -0.39 – 1.55 | 0.242 |
| random effects |  |  |  |
| σ2 | 1.88 |  |  |
| τ00 VP | 1.65 |  |  |
| ICC | 0.47 |  |  |
| N VP | 126 |  |  |
| Observations | 494 |  |  |
| marginal R2 / conditional R2 | 0.377 / 0.669 |  |  |

*Note.* ROI = region of interest, HC = healthy controls, EA = subjective emotional arousal at time point 2 with EA 1 serving as the reference category, CI = confidence interval, ICC = intraclass correlation coefficient.

The analyses of the Cyberball manipulation check (Table 2S) in the HC group revealed significant main effects of condition for all NTS subscales (belonging: b = 20.06, *p* < 0.001; control: b = 23.68, *p* < 0.001; self: b = 10.06, *p* = 0.015; meaning: b = 13.96, *p* < 0.001), with higher levels in the control condition than in the stress condition, i.e., less need threat in the control vs. the stress condition. The analysis revealed a significant influence of the study day only for the belonging subscale (b = 9.41, *p* = 0.001).

Table S2. Linear mixed models of NTS subscales of HC group for Cyberball manipulation check.

| NTS belonging | Predictors | Estimates | CI | *p* |
| --- | --- | --- | --- | --- |
|  | **(Intercept)** | **17.75** | **12.61 – 22.89** | **<0.001** |
|  | **condition [CONTROL]** | **20.06** | **14.43 – 25.69** | **<0.001** |
|  | **study day [2]** | **9.41** | **3.78 – 15.04** | **0.001** |
|  | random effects |  |  |  |
|  | σ2 | 227.91 |  |  |
|  | τ00 VP | 51.50 |  |  |
|  | ICC | 0.18 |  |  |
|  | N VP | 58 |  |  |
|  | observations | 114 |  |  |
|  | marginal R2 / conditional R2 | 0.318 / 0.444 |  |  |
| NTS control | Predictors | Estimates | CI | *p* |
|  | **(Intercept)** | **20.37** | **14.56 – 26.17** | **<0.001** |
|  | **condition [CONTROL]** | **23.67** | **16.84 – 30.50** | **<0.001** |
|  | study day [2] | -4.36 | -11.20 – 2.47 | 0.208 |
|  | random effects |  |  |  |
|  | σ2 | 336.80 |  |  |
|  | τ00 VP | 0.00 |  |  |
|  | N VP | 58 |  |  |
|  | observations | 114 |  |  |
|  | marginal R2 / conditional R2 | 0.297 / NA |  |  |
| NTS self | Predictors | Estimates | CI | *p* |
|  | **(Intercept)** | **36.47** | **29.60 – 43.34** | **<0.001** |
|  | **condition [CONTROL]** | **10.06** | **1.97 – 18.15** | **0.015** |
|  | study day [2] | 0.86 | -7.23 – 8.95 | 0.834 |
|  | random effects |  |  |  |
|  | σ2 | 472.23 |  |  |
|  | τ00 VP | 0.00 |  |  |
|  | N VP | 58 |  |  |
|  | observations | 114 |  |  |
|  | marginal R2 / conditional R2 | 0.052 / NA |  |  |
| NTS meaning | Predictors | Estimates | CI | *p* |
|  | **(Intercept)** | **21.63** | **15.83 – 27.43** | **<0.001** |
|  | **condition [CONTROL]** | **13.96** | **7.13 – 20.78** | **<0.001** |
|  | study day [2] | 2.09 | -4.74 – 8.91 | 0.546 |
|  | random effects |  |  |  |
|  | σ2 | 336.34 |  |  |
|  | τ00 VP | 0.00 |  |  |
|  | N VP | 58 |  |  |
|  | observations | 114 |  |  |
|  | marginal R2 / conditional R2 | 0.132 / NA |  |  |

*Note.* NTS = Need Threat Scale, CI = confidence interval.

S3.2. Physiological data

S3.2.1 *EDA*

Average scores on the EDA measures are displayed in Table S3. The LMM model comparison revealed that the complex model did not fit the data better than the simpler model (χ²(3) = 3.304, *p* = 0.347), while none of the covariates had a significant impact on the model (all *p* > 0.132). Results for the simpler LMM of EDA levels are presented in detail in Table S4.

The model of EDA measures revealed a significant three-way interaction between ROI, condition, and group for the TSSTm (b = 3.67, *p* = 0.006). All participants showed a significant decrease in EDA over the two study days (b = -0.79, *p* = 0.001).

Figure S1 shows the predicted values of EDA measures for both groups over the four ROIs and both conditions.

Table S3. Mean EDA measures [µS] and standard deviation of BPD and HC group for each ROI.

|  |  | BPD |  | HC |  |
| --- | --- | --- | --- | --- | --- |
| ROI | condition | n | M (SD) | n | M (SD) |
| Acc | control | 51 | 2.80 (3.17) | 51 | 2.27 (2.28) |
| Acc | stress | 36 | 1.41 (1.51) | 44 | 1.59 (1.93) |
| TSSTs | control | 52 | 5.93 (7.16) | 50 | 3.02 (3.33) |
| TSSTs | stress | 38 | 2.65 (3.35) | 51 | 3.15 (3.16) |
| TSSTm | control | 52 | 9.35 (10.20) | 49 | 4.35 (4.44) |
| TSSTm | stress | 37 | 3.88 (4.54) | 46 | 3.90 (3.04) |
| Cyberball | control | 48 | 5.09 (5.18) | 47 | 3.84 (3.07) |
| Cyberball | stress | 42 | 5.75 (7.13) | 47 | 3.67 (3.38) |

*Note.* EDA = electrodermal activity, ROI = region of interest, Acc = Acclimatization, TSSTs = Trier Social Stress Test speech task, TSSTm = Trier Social Stress Test mental arithmetic task, BPD = patients with borderline personality disorder symptoms, HC = healthy controls, M = mean, SD = standard deviation.

Table S4. Linear mixed model of EDA.

| Predictors | Estimates | CI | *p* |
| --- | --- | --- | --- |
| **(Intercept)** | **4.11** | **2.57 – 5.65** | **<0.001** |
| **ROI [TSSTs]** | **3.19** | **1.98 – 4.40** | **<0.001** |
| **ROI [TSSTm]** | **6.61** | **5.40 – 7.81** | **<0.001** |
| **ROI [Cyberball]** | **3.07** | **1.83 – 4.31** | **<0.001** |
| condition [STRESS] | -0.96 | -2.34 – 0.42 | 0.171 |
| group [HC] | -0.67 | -2.59 – 1.25 | 0.492 |
| **study day** | **-0.79** | **-1.28 – -0.30** | **0.001** |
| ROI [TSSTs] × condition [STRESS] | -1.49 | -3.37 – 0.40 | 0.122 |
| **ROI [TSSTm] × condition [STRESS]** | **-3.42** | **-5.31 – -1.53** | **<0.001** |
| ROI [Cyberball] × condition [STRESS] | 1.04 | -0.84 – 2.93 | 0.278 |
| **ROI [TSSTs] × group [HC]** | **-2.30** | **-4.03 – -0.58** | **0.009** |
| **ROI [TSSTm] × group [HC]** | **-4.28** | **-6.01 – -2.54** | **<0.001** |
| ROI [Cyberball] × group [HC] | -1.37 | -3.14 – 0.40 | 0.128 |
| cond [STRESS] × group [HC] | 0.31 | -1.59 – 2.20 | 0.752 |
| (ROI [TSSTs] × condition [STRESS]) × group [HC] | 2.13 | -0.47 – 4.72 | 0.108 |
| **(ROI [TSSTm] × condition [STRESS]) × group [HC]** | **3.67** | **1.05 – 6.29** | **0.006** |
| (ROI [Cyberball] × condition [STRESS]) × group [HC] | -0.52 | -3.14 – 2.09 | 0.695 |
| random effects |  |  |  |
| σ2 | 9.93 |  |  |
| τ00 VP | 15.89 |  |  |
| ICC | 0.62 |  |  |
| N VP | 114 |  |  |
| observations | 745 |  |  |
| marginal R2 / conditional R2 | 0.137 / 0.668 |  |  |

*Note.* EDA = electrodermal activity, ROI = region of interest, HC = healthy controls, TSSTs = Trier Social Stress Test speech task, TSSTm = Trier Social Stress Test mental arithmetic task, ICC = intraclass correlation coefficient, CI = confidence interval.


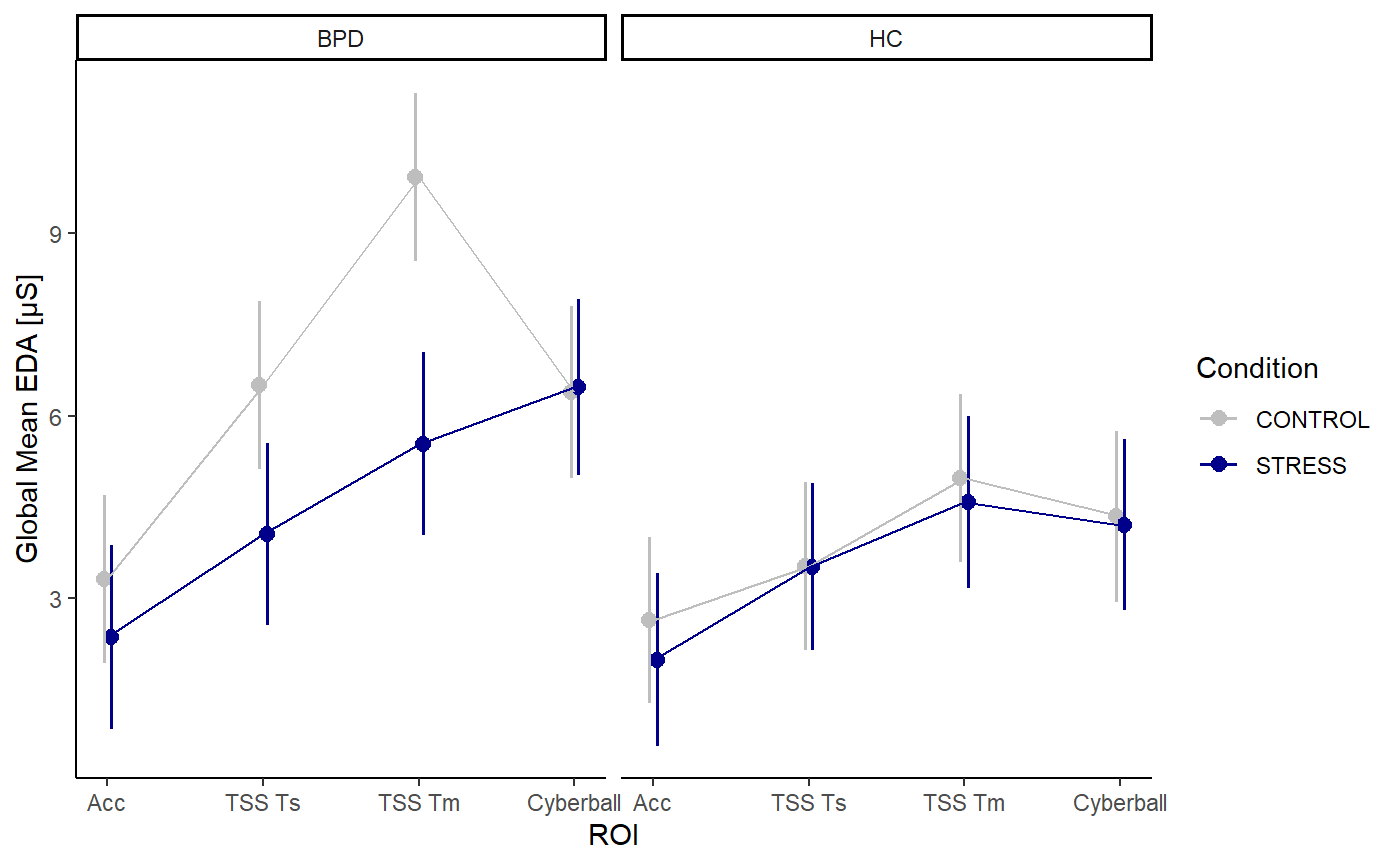
 Figure S1. Predicted values of the linear mixed model of electrodermal activity (EDA) [µS].

*Note.* Acc = Acclimatization, TSSTs/m = Trier Social Stress Test speech task/mental arithmetic task, BPD = patients with borderline personality disorder symptoms, HC = healthy controls, ROI = region of interest.

S3.2.2. *Cortisol*

Average scores for cortisol levels are displayed in Table S5. The LMM model comparison revealed that the complex model did not fit the data better than the simpler model (χ²(3) = 4.3244, *p* = 0.229), while none of the covariates had a significant impact on the model (all *p* > 0.180). Results for the simpler LMM of cortisol levels are presented in detail in Table S6.

The model of cortisol showed significant three-way interactions between ROI, condition, and group for Cyberball (b = 1.02, *p* = 0.005), VRQuestionnaires (b = 0.86, *p* = 0.020), and post (b = 0.80, *p* = 0.029). These interactions depict different cortisol patterns between the groups: While the BPD group showed a significant decrease in cortisol at the ROIs Cyberball, VRQuestionnaires, and post under stress compared to the control condition, the HC group showed more stable cortisol patterns at these ROIs, with a slight tendency towards higher cortisol under stress. This difference was particularly pronounced in Cyberball, in which the difference between the stress and control conditions was greatest in the BPD group.

Figure S2 shows the predicted values of cortisol levels for both groups over the six ROIs and both conditions.

Table S5. Mean cortisol level [nmol/l] and standard deviation of BPD and HC group for each ROI.

|  |  | BPD |  | HC |  |
| --- | --- | --- | --- | --- | --- |
| ROI | condition | n | M (SD) | n | M (SD) |
| prestart | control | 42 | 3.04 (1.33) | 53 | 2.94 (1.48) |
| prestart | stress | 46 | 3.11 (1.54) | 50 | 2.63 (0.95) |
| Acc | control | 43 | 3.11 (1.34) | 54 | 3.03 (1.55) |
| Acc | stress | 45 | 3.18 (1.70) | 51 | 2.60 (0.90) |
| TSST | control | 45 | 3.16 (1.52) | 53 | 2.83 (1.46) |
| TSST | stress | 45 | 2.85 (1.35) | 53 | 2.75 (1.21) |
| Cyberball | control | 46 | 3.10 (1.55) | 51 | 2.52 (1.19) |
| Cyberball | stress | 45 | 2.49 (1.12) | 53 | 2.79 (1.37) |
| VRQuestionnaires | control | 45 | 2.77 (1.33) | 51 | 2.33 (1.08) |
| VRQuestionnaires | stress | 42 | 2.27 (0.93) | 51 | 2.52 (1.05) |
| post | control | 43 | 2.70 (1.32) | 49 | 2.27 (1.02) |
| post | stress | 46 | 2.48 (1.27) | 51 | 2.59 (1.10) |

*Note.* ROI = region of interest, Acc = Acclimatization, TSST = Trier Social Stress Test, BPD = patients with borderline personality disorder symptoms, HC = healthy controls.

Table S6. Linear mixed model of cortisol levels.

| Predictors | Estimates | CI | *p* |
| --- | --- | --- | --- |
| **(Intercept)** | **3.05** | **2.63 – 3.47** | **<0.001** |
| ROI [Acc] | 0.11 | -0.27 – 0.49 | 0.579 |
| ROI [TSST] | 0.11 | -0.27 – 0.49 | 0.569 |
| ROI [Cyberball] | 0.02 | -0.36 – 0.39 | 0.937 |
| ROI [VRQuestionnaires] | -0.32 | -0.70 – 0.05 | 0.092 |
| **ROI [post]** | **-0.38** | **-0.76 – -0.00** | **0.049** |
| cond [STRESS] | 0.02 | -0.36 – 0.39 | 0.929 |
| group [HC] | -0.18 | -0.70 – 0.35 | 0.502 |
| day | 0.06 | -0.04 – 0.17 | 0.231 |
| ROI [Acc] × cond [STRESS] | -0.03 | -0.55 – 0.50 | 0.926 |
| ROI [TSST] × cond [STRESS] | -0.37 | -0.89 – 0.16 | 0.174 |
| **ROI [Cyberball] × cond [STRESS]** | **-0.62** | **-1.14 – -0.09** | **0.021** |
| ROI [VRQuestionnaires] × cond [STRESS] | -0.43 | -0.96 – 0.10 | 0.110 |
| ROI [post] × cond [STRESS] | -0.27 | -0.80 – 0.25 | 0.309 |
| ROI [Acc] × group [HC] | -0.03 | -0.54 – 0.48 | 0.899 |
| ROI [TSST] × group [HC] | -0.21 | -0.72 – 0.30 | 0.411 |
| ROI [Cyberball] × group [HC] | -0.36 | -0.87 – 0.14 | 0.160 |
| ROI [VRQuestionnaires] × group [HC] | -0.22 | -0.73 – 0.29 | 0.400 |
| ROI [post] × group [HC] | -0.20 | -0.72 – 0.31 | 0.439 |
| cond [STRESS] × group [HC] | -0.20 | -0.72 – 0.31 | 0.436 |
| (ROI [Acc] × cond [STRESS]) × group [HC] | -0.09 | -0.81 – 0.63 | 0.804 |
| (ROI [TSST] × cond [STRESS]) × group [HC] | 0.55 | -0.17 – 1.27 | 0.132 |
| **(ROI [Cyberball] × cond [STRESS]) × group [HC]** | **1.02** | **0.31 – 1.74** | **0.005** |
| **(ROI [VRQuestionnaires] × cond [STRESS]) × group [HC]** | **0.86** | **0.13 – 1.58** | **0.020** |
| **(ROI [post] × cond [STRESS]) × group [HC]** | **0.80** | **0.08 – 1.53** | **0.029** |
| random effects |  |  |  |
| σ2 | 0.80 |  |  |
| τ00 VP | 0.98 |  |  |
| ICC | 0.55 |  |  |
| N VP | 107 |  |  |
| observations | 1153 |  |  |
| marginal R2 / conditional R2 | 0.038 / 0.568 |  |  |

*Note.* ROI = region of interest, Acc = Acclimatization, TSST = Trier Social Stress Test speech task, HR = healthy controls, ICC = intraclass correlation coefficient, CI = confidence interval.


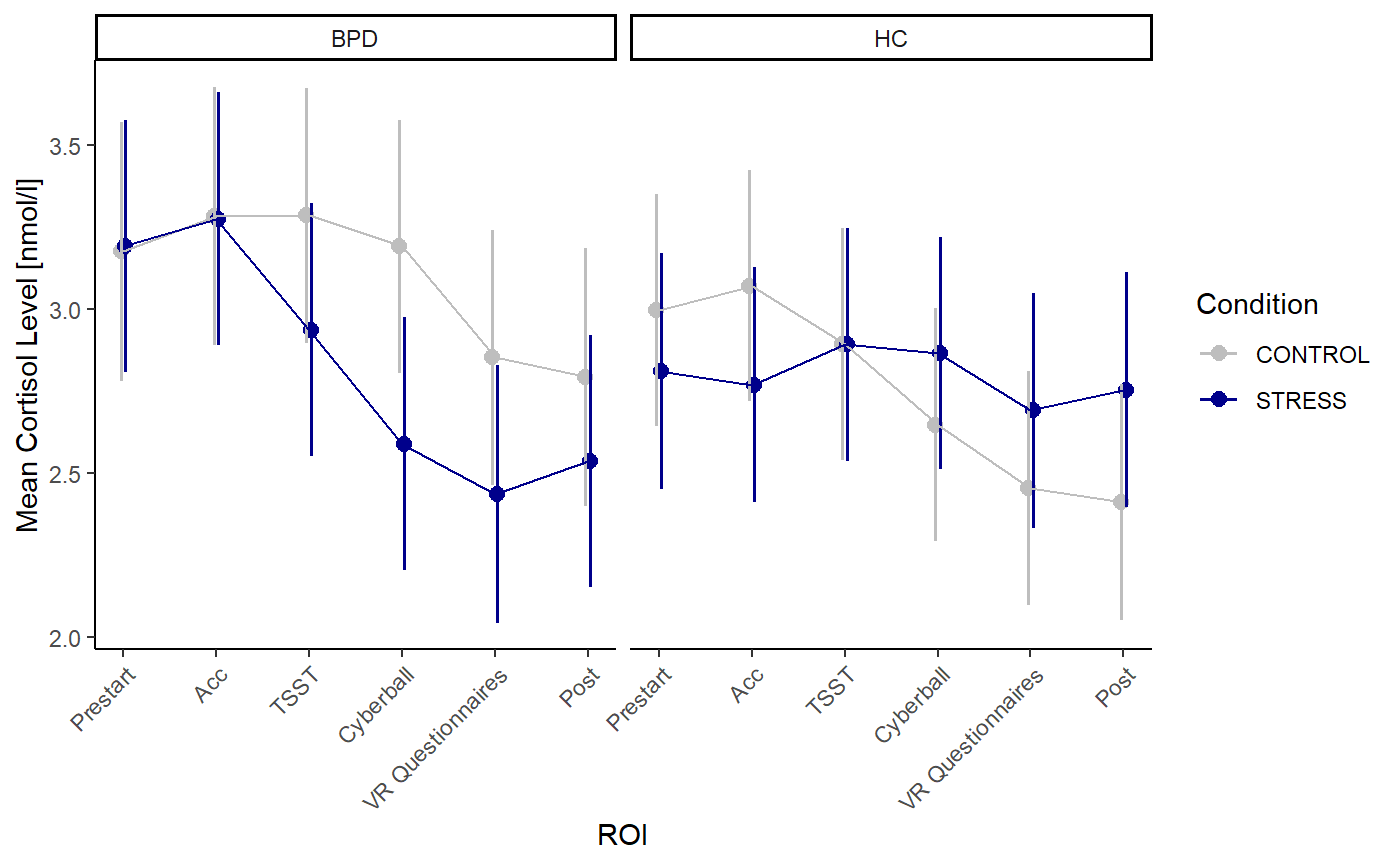
Figure S2. Predicted values of the linear mixed model of cortisol levels [nmol/l].

*Note.* Acc = Acclimatization, TSST = Trier Social Stress Test, BPD = patients with borderline personality disorder symptoms, HC = healthy controls, ROI = region of interest.


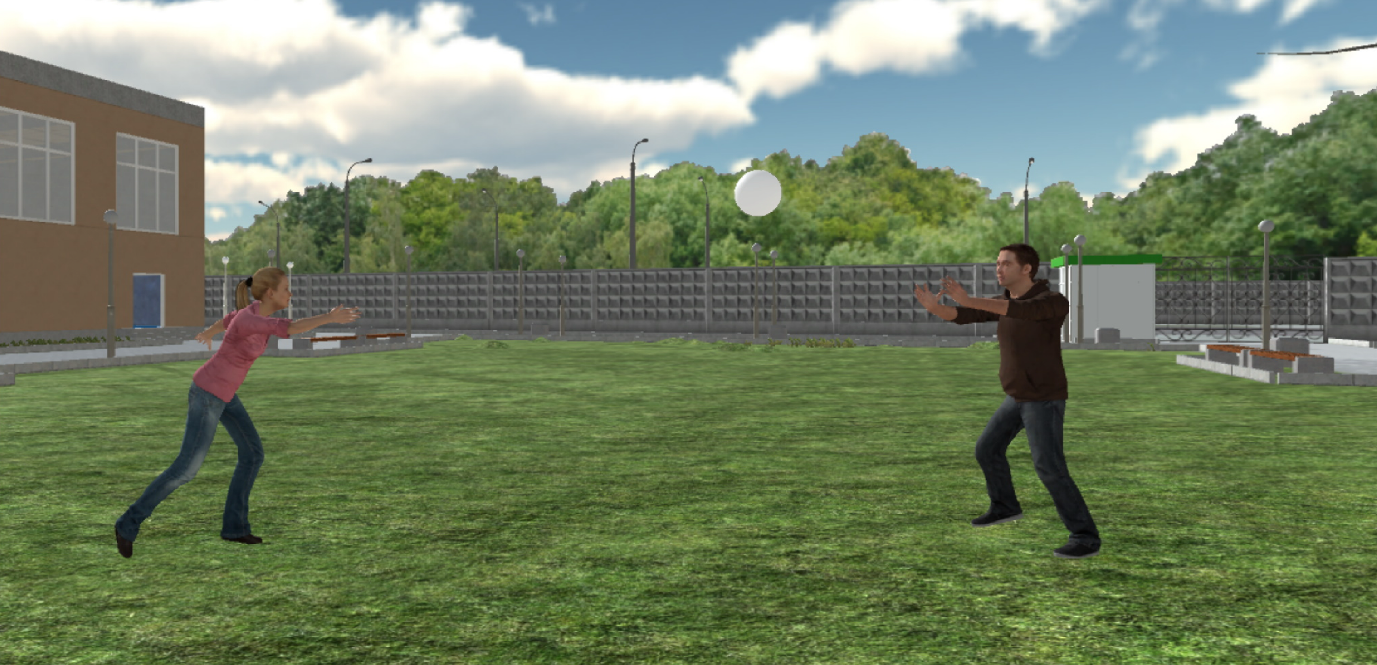


Figure S3. Cyberball in *aVeRsive tension*.
