# Supplementary material for: aVeRsive tension: A new virtual reality paradigm to assess emotional arousal in adolescent and young adult patients with symptoms of borderline personality disorder
Source: Int J Clin Health Psychol. 2025 May 5;25(2):100583. doi: 10.1016/j.ijchp.2025.100583 (PMC12138408; doi:10.1016/j.ijchp.2025.100583)
Supplement: Supplementary file 2 [file mmc2.docx]

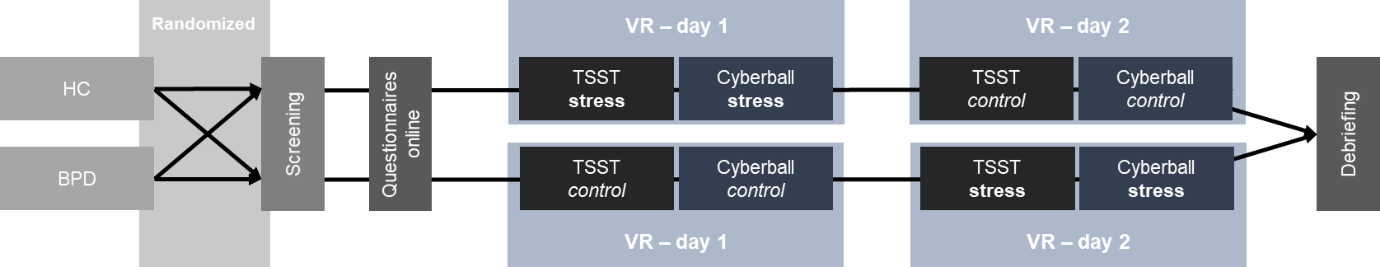


Figure 1. Study design. HC = healthy control participants, BPD = participants with borderline personality disorder symptoms, VR = virtual reality, TSST = Trier Social Stress Test.


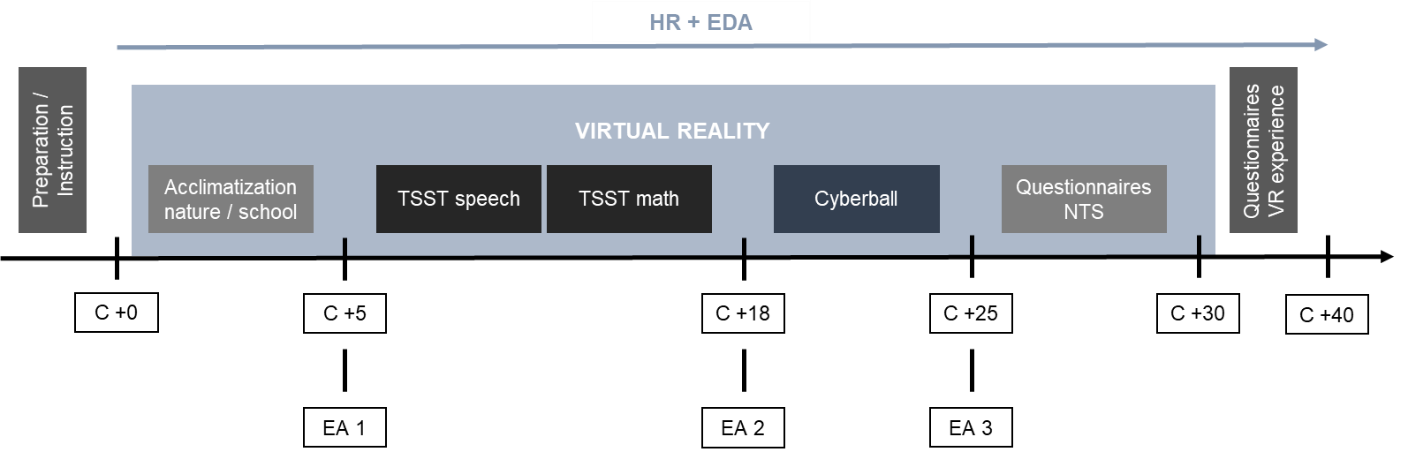


Figure 2. Experimental procedure and measures. HR = heart rate, EDA = electrodermal activity, TSST = Trier Social Stress Test, NTS = Need Threat Scale, C +minutes = cortisol measures at 6 time points, EA 1-3 = subjective emotional arousal rating.


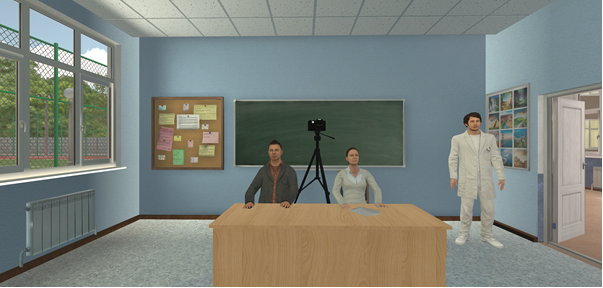


Figure 3. Trier Social Stress Test (TSST) in *aVeRsive tension*.

Table 1. Sample characteristics and group comparisons.

|  | | BPD |  |  | HC |  |  | Group comparisons |  |  |
| --- | --- | --- | --- | --- | --- | --- | --- | --- | --- | --- |
| n | | 62 |  |  | 62 |  |  |  |  |  |
|  | | female | male | diverse | female | male | diverse | Fisher’s exact test |  | *p* |
| **gender n (%)** | | 50 (80.65%) | 5 (8.06%) | 7 (11.29%) | 59 (95.16%) | 2 (3.23%) | 1 (1.61%) |  |  | **0.032** |
|  | |  | M | SD |  | M | SD | t | df | *p* |
| age in years | |  | 18.11 | 2.68 |  | 18.21 | 2.73 | -0.199 | 122 | 0.842 |
| BPD criteria | |  | 5.42 | 1.83 |  |  |  |  |  |  |
| current treatment n (%) | | 46 (74.19%) |  |  | 0 |  |  |  |  |  |
| medication | |  |  |  |  |  |  |  |  |  |
| at least one psychotropic medication n (%) | | 39 (62.9%) |  |  | 0 |  |  |  |  |  |
| *antidepressants** | | 31 (50%) |  |  |  |  |  |  |  |  |
| *neuroleptics* | | 24 (38.7%) |  |  |  |  |  |  |  |  |
| *stimulants* | | 8 (12.9%) |  |  |  |  |  |  |  |  |
| *sleep medication* | | 6 (9.7%) |  |  |  |  |  |  |  |  |
| Patients with multiple medication classes | | 21 (33.9%) |  |  |  |  |  |  |  |  |
|  | |  |  |  |  |  |  | Welch t-test |  |  |
|  | | n | M | SD | n | M | SD | t | df | *p* |
| **DSS** | | 59 | 3.55 | 2.49 | 61 | 0.38 | 0.96 | 9.157 | 74.479 | **< 0.001** |
| **BSCL** | | 60 | 26.99 | 6.12 | 61 | 12.9 | 3.03 | 16.006 | 86.029 | **< 0.001** |
| **SPAIC** | | 58 | 30.83 | 12.21 | 61 | 11.61 | 8.27 | 10.004 | 99.573 | **< 0.001** |
| **NTS** | |  |  |  |  |  |  |  |  |  |
| *belonging* | **stress** | 53 | 2.14 | 0.82 | 57 | 2.74 | 0.98 | -3.503 | 106.81 | **< 0.001** |
|  | **control** | 54 | 4.04 | 1.23 | 57 | 5.3 | 0.77 | -6.451 | 88.237 | **< 0.001** |
| *control* | stress | 53 | 1.82 | 0.85 | 57 | 2.16 | 1.1 | -1.831 | 104.39 | 0.070 |
|  | **control** | 54 | 4.69 | 1.36 | 57 | 5.29 | 0.99 | -2.625 | 96.741 | **0.010** |
| *self* | **stress** | 53 | 3.15 | 1.58 | 57 | 4.48 | 1.21 | -4.92 | 97.085 | **<0.001** |
|  | **control** | 54 | 4.39 | 1.62 | 57 | 6.01 | 0.99 | -6.343 | 86.733 | **<0.001** |
| *meaning* | **stress** | 53 | 1.77 | 0.88 | 57 | 2.85 | 1.29 | -5.139 | 99.368 | **<0.001** |
|  | **control** | 54 | 3.59 | 1.49 | 57 | 4.98 | 1.03 | -5.723 | 93.854 | **<0.001** |
| **FMSQ** | |  |  |  |  |  |  |  |  |  |
|  | **stress** | 55 | 5.51 | 4.58 | 60 | 2.5 | 2.37 | 4.365 | 79.337 | **< 0.001** |
|  | **control** | 57 | 6.02 | 5.2 | 58 | 2.55 | 2.66 | 4.484 | 83.124 | **< 0.001** |
| PRE | |  |  |  |  |  |  |  |  |  |
|  | stress | 51 | 5.18 | 2.35 | 53 | 5.02 | 1.81 | 0.382 | 94.088 | 0.703 |
|  | control | 53 | 5.38 | 2.47 | 53 | 4.87 | 1.83 | 1.205 | 95.771 | 0.231 |

*Note.* M = mean, SD = standard deviation, BPD = patients with borderline personality disorder symptoms, HC = healthy controls, BSCL = Brief Symptom Checklist, DSS = Dissociation Tension Scale acute, * Percentages sum to more than 100% due to patients taking multiple medication classes, DSS-acute SPAIC = Social Phobia and Anxiety Inventory for Children, NTS = Need Threat Scale, FMSQ = Fast Motion Sickness Questionnaire, PRE = Presence

Table 2. Mean outcome measures of subjective EA for the BPD and HC groups at all ROIs.

|  | | BPD | | HC | |
| --- | --- | --- | --- | --- | --- |
| ROI | condition | n | M (SD) | n | M (SD) |
| EA 1 | control | 61 | 4.43 (1.81) | 64 | 2.22 (1.70) |
| EA 1 | stress | 57 | 4.33 (1.86) | 65 | 2.20 (1.48) |
| EA 2 | control | 61 | 6.16 (2.09) | 64 | 3.11 (2.18) |
| EA 2 | stress | 57 | 6.02 (2.12) | 65 | 3.62 (2.07) |
| EA 3 | control | 59 | 4.90 (2.16) | 61 | 1.79 (1.03) |
| EA 3 | stress | 56 | 5.29 (2.13) | 62 | 2.50 (1.56) |

*Note.* ROI = region of interest, EA 1-3 = subjective emotional arousal at time points 1-3, BPD = patients with borderline personality disorder symptoms, HC = healthy controls.

Table 3. Linear mixed model of subjective EA.

| Predictors | Estimates | CI | *p* |
| --- | --- | --- | --- |
| **(Intercept)** | **5.37** | **4.83 – 5.90** | **<0.001** |
| **ROI [EA 2]** | **1.76** | **1.26 – 2.25** | **<0.001** |
| ROI [EA 3] | 0.49 | -0.01 – 0.99 | 0.054 |
| condition [STRESS] | -0.11 | -0.62 – 0.40 | 0.682 |
| **group [HC]** | **-2.26** | **-2.88 – -1.64** | **<0.001** |
| zage | 0.04 | -0.17 – 0.26 | 0.692 |
| **zSPAIC** | **0.49** | **0.27 – 0.72** | **<0.001** |
| **zDSS** | **0.39** | **0.16 – 0.62** | **0.001** |
| **study day** | **-0.62** | **-0.83 – -0.42** | **<0.001** |
| ROI [EA 2] × condition [STRESS] | -0.04 | -0.76 – 0.68 | 0.909 |
| ROI [EA 3] × condition [STRESS] | 0.51 | -0.21 – 1.23 | 0.168 |
| **ROI [EA 2] × group [HC]** | **-0.79** | **-1.48 – -0.10** | **0.025** |
| **ROI [EA 3] × group [HC]** | **-0.92** | **-1.62 – -0.22** | **0.010** |
| condition [STRESS] × group [HC] | 0.11 | -0.59 – 0.81 | 0.757 |
| (ROI [EA 2] × condition [STRESS]) × group [HC] | 0.51 | -0.48 – 1.50 | 0.312 |
| (ROI [EA 3] × condition [STRESS]) × group [HC] | 0.22 | -0.78 – 1.22 | 0.668 |
| random effects |  |  |  |
| σ^2^ | 1.85 |  |  |
| τ00 VP | 1.09 |  |  |
| ICC | 0.37 |  |  |
| N VP | 119 |  |  |
| observations | 693 |  |  |
| marginal R^2^ / conditional R^2^ | 0.493 / 0.680 |  |  |

*Note.* CI = confidence interval, ROI = region of interest, EA 1-3 = subjective emotional arousal at time points 1-3, with EA 1 serving as the reference category, HC = healthy controls, zSPAIC = z-standardized values of Social Phobia and Anxiety Inventory for Children, zDSS = z-standardized scores of dissociation tendency, ICC = intraclass correlation coefficient.


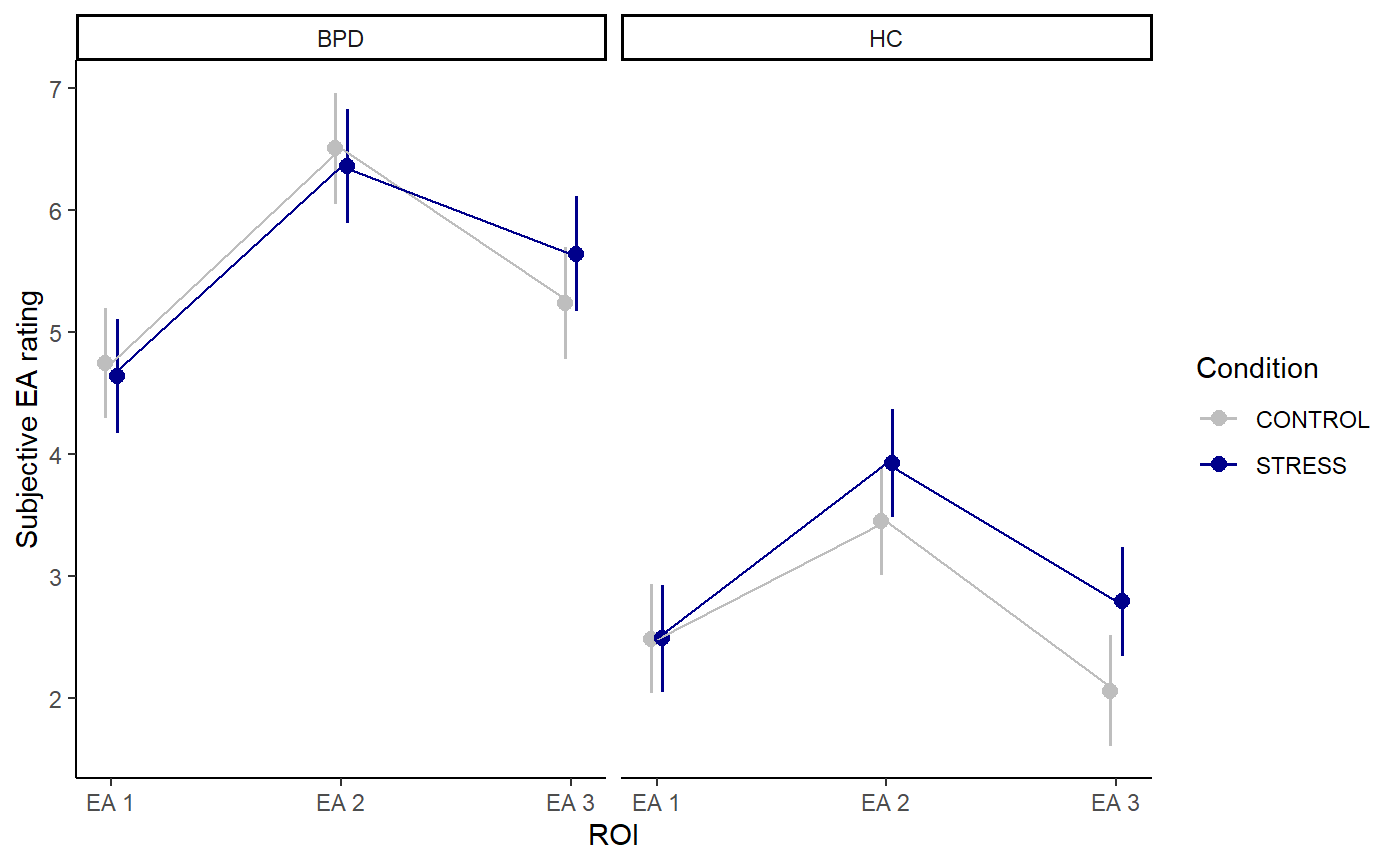


Figure 4. Predicted values of the linear mixed model of EA.
*Note.* EA 1-3 = emotional arousal at (1) baseline, (2) after TSST, and (3) after Cyberball, BPD = patients with borderline personality disorder symptoms, HC = healthy controls, ROI = region of interest.

Table 4. Pairwise comparisons of EA 2 and EA 3 per group and condition.

| contrast |  | estimate | SE | df | z-ratio | *Bonferroni adj. p* |
| --- | --- | --- | --- | --- | --- | --- |
| **Control BPD EA 2** | **Control BPD EA 3** | **1.266** | **0.255** | **564** | **4.962** | **< 0.001** |
| Stress BPD EA 2 | Stress BPD EA 3 | 0.716 | 0.266 | 564 | 2.694 | 0.232 |
| **Control HC EA 2** | **Control HC EA 3** | **1.393** | **0.250** | **564** | **5.575** | **< 0.001** |
| **Stress HC EA 2** | **Stress HC EA 3** | **1.134** | **0.248** | **564** | **4.574** | **< 0.001** |

BPD = patients with borderline personality disorder symptoms, HC = healthy controls, SE = standard error, df = degrees of freedom, Bonferroni-adjusted *p*-value = 0.0125.

Table 5. Mean heart rate [Hz] and standard deviation of BPD and HC group for each ROI.

|  |  | BPD |  | HC |  |
| --- | --- | --- | --- | --- | --- |
| ROI | condition | n | M (SD) | n | M (SD) |
| Acc | control | 54 | 90.13 (15.60) | 50 | 84.53 (12.36) |
| Acc | stress | 44 | 87.97 (14.05) | 48 | 85.61 (12.76) |
| TSSTs | control | 55 | 92.84 (17.51) | 51 | 87.24 (13.95) |
| TSSTs | stress | 48 | 94.49 (15.87) | 53 | 89.22 (14.61) |
| TSSTm | control | 55 | 90.63 (17.90) | 51 | 86.88 (15.87) |
| TSSTm | stress | 47 | 91.02 (16.48) | 53 | 89.65 (19.22) |
| Cyberball | control | 55 | 90.51 (16.64) | 50 | 89.00 (14.87) |
| Cyberball | stress | 49 | 92.44 (17.04) | 53 | 86.55 (13.80) |

*Note.* HR = heart rate, ROI = region of interest, Acc = Acclimatization, TSSTs = Trier Social Stress Test speech task, TSSTm = Trier Social Stress Test mental arithmetic task, BPD = patients with borderline personality disorder symptoms, HC = healthy controls, M = mean, SD = standard deviation.

Table 6. Linear mixed model of HR.

| Predictors | Estimates | CI | *p* |
| --- | --- | --- | --- |
| **(Intercept)** | **89.72** | **88.06 – 91.38** | **<0.001** |
| **ROI [TSSTs]** | **3.66** | **3.35 – 3.97** | **<0.001** |
| **ROI [TSSTm]** | **1.48** | **1.17 – 1.79** | **<0.001** |
| **ROI [Cyberball]** | **1.38** | **1.07 – 1.69** | **<0.001** |
| **condition [STRESS]** | **-2.51** | **-2.86 – -2.17** | **<0.001** |
| **group [HC]** | **-4.17** | **-5.26 – -3.07** | **<0.001** |
| **study day** | **-0.24** | **-0.35 – -0.12** | **<0.001** |
| **ROI [TSSTs] × condition [STRESS]** | **4.03** | **3.56 – 4.49** | **<0.001** |
| **ROI [TSSTm] × condition [STRESS]** | **2.89** | **2.43 – 3.35** | **<0.001** |
| **ROI [Cyberball] × condition [STRESS]** | **4.04** | **3.60 – 4.49** | **<0.001** |
| **ROI [TSSTs] × group [HC]** | **-1.64** | **-2.10 – -1.18** | **<0.001** |
| ROI [TSSTm] × group [HC] | 0.19 | -0.27 – 0.65 | 0.417 |
| **ROI [Cyberball] × group [HC]** | **2.02** | **1.57 – 2.47** | **<0.001** |
| **condition [STRESS] × group [HC]** | **3.33** | **2.84 – 3.82** | **<0.001** |
| **(ROI [TSSTs] × condition [STRESS]) × group [HC]** | **-2.46** | **-3.11 – -1.80** | **<0.001** |
| (ROI [TSSTm] × condition [STRESS]) × group [HC] | -0.57 | -1.22 – 0.09 | 0.090 |
| **(ROI [Cyberball] × condition [STRESS]) × group [HC]** | **-6.48** | **-7.11 – -5.84** | **<0.001** |
| random effects |  |  |  |
| σ2 | 185.45 |  |  |
| τ00 VP | 70.84 |  |  |
| ICC | 0.28 |  |  |
| N VP | 113 |  |  |
| observations | 240506 |  |  |
| marginal R2 / conditional R2 | 0.024 / 0.294 |  |  |

*Note.* HR = heart rate, ROI = region of interest, HR = healthy controls, TSSTs = Trier Social Stress Test speech task, TSSTm = Trier Social Stress Test mental arithmetic task, ICC = intraclass correlation coefficient, CI = confidence interval.


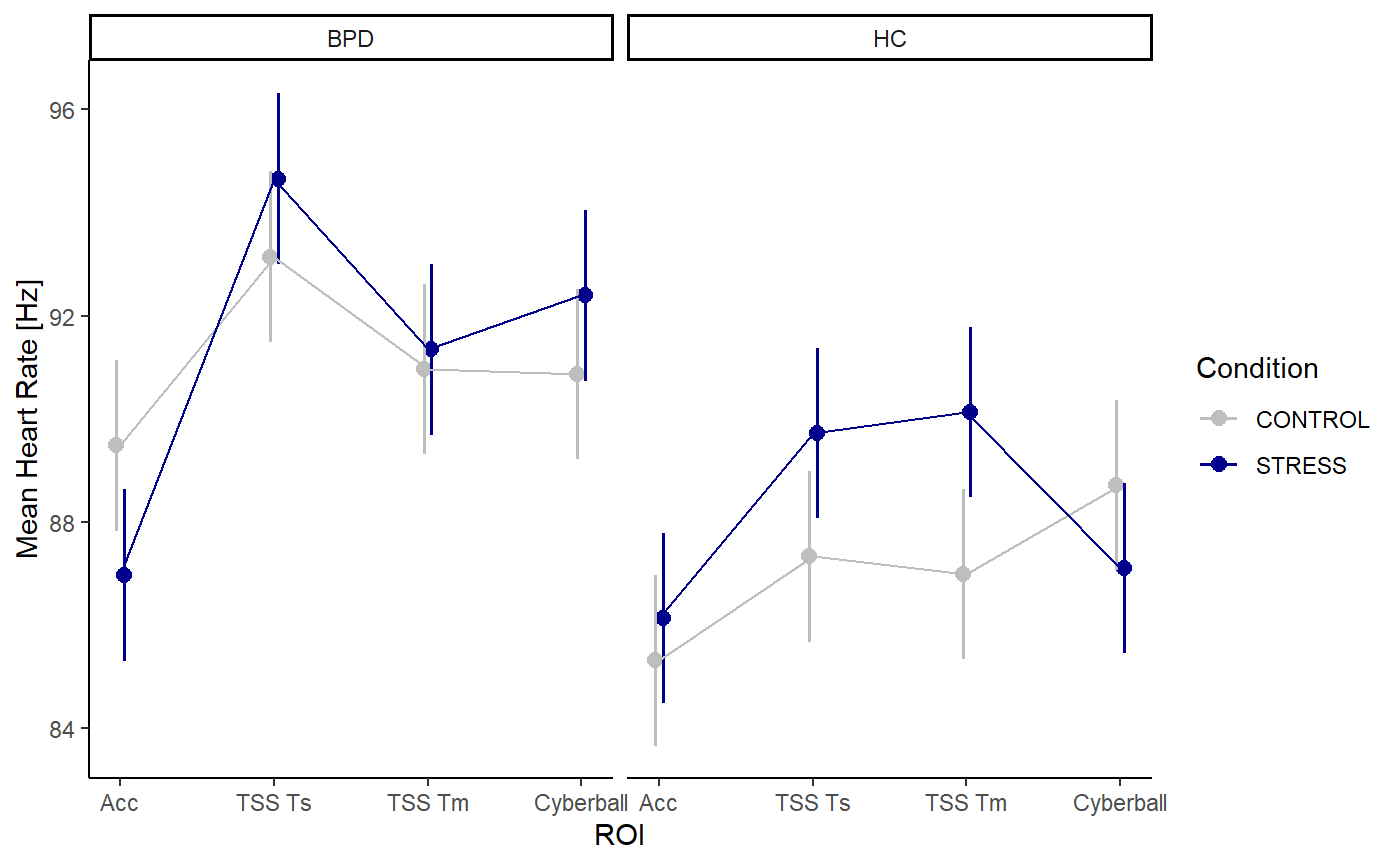


Figure 5. Predicted values of the linear mixed model of heart rate [Hz].

*Note.* Acc = Acclimatization, TSSTs/m = Trier Social Stress Test speech task/mental arithmetic task, BPD = patients with borderline personality disorder symptoms, HC = healthy controls, ROI = region of interest.
